# Fungal Anthraquinone Photoantimicrobials Challenge the Dogma of Cationic Photosensitizers

**DOI:** 10.1021/acs.jnatprod.2c01157

**Published:** 2023-09-14

**Authors:** Fabian Hammerle, Johannes Fiala, Anja Höck, Lesley Huymann, Pamela Vrabl, Yurii Husiev, Sylvestre Bonnet, Ursula Peintner, Bianka Siewert

**Affiliations:** † Department of Department of Pharmacognosy, Institute of Pharmacy, CCB − Centrum of Chemistry and Biomedicine, CMBI − Center for Molecular Biosciences, University of Innsbruck, 6020 Innsbruck, Austria; ‡ Institute of Microbiology, University of Innsbruck, 6020 Innsbruck, Austria; § Leiden Institute of Chemistry, Leiden University, 2333CC Leiden, The Netherlands; △ Department of Biotechnology & Food Engineering, MCI-The Entrepreneurial School, 6020 Innsbruck, Austria

## Abstract

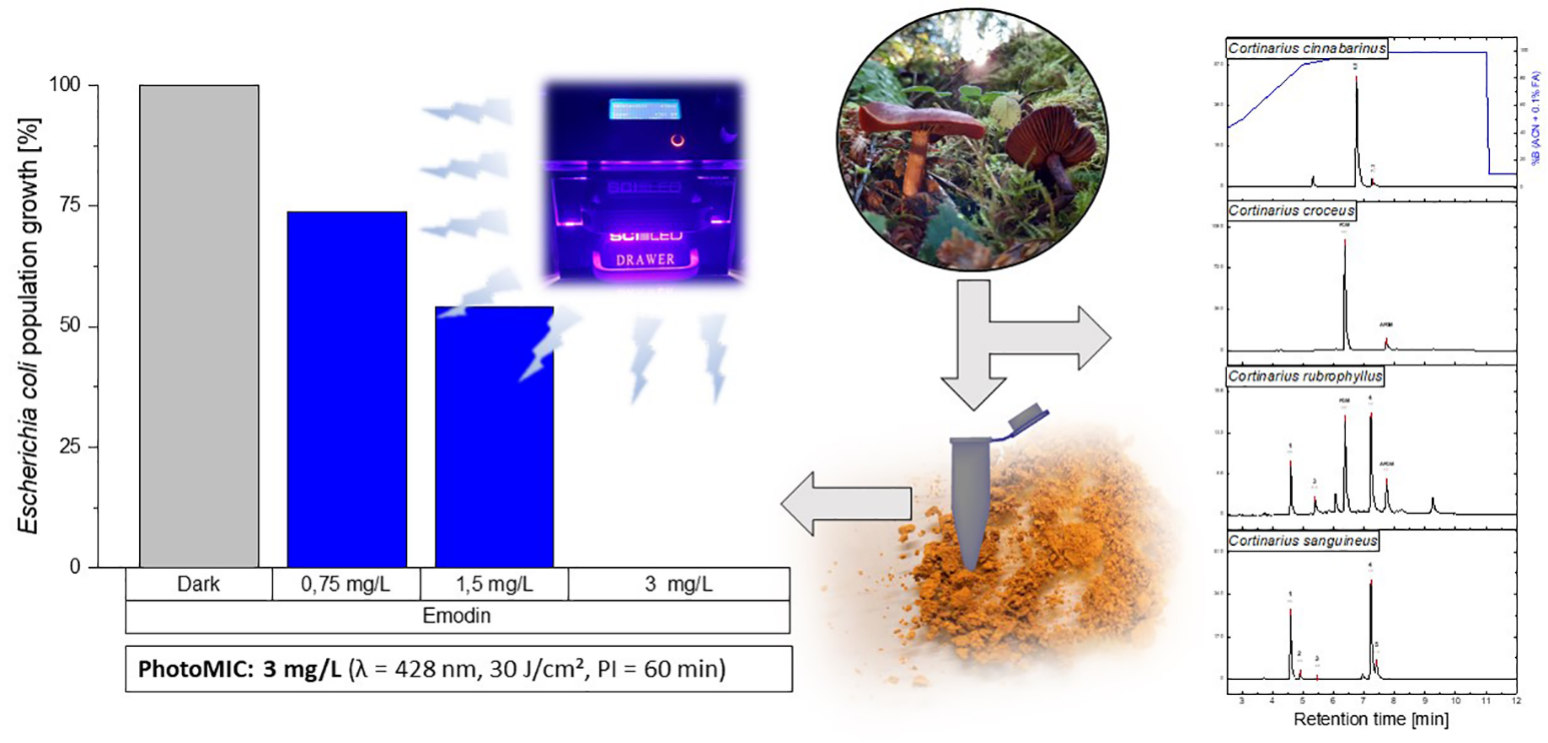

The photoantimicrobial potential of four mushroom species (i.e., *Cortinarius cinnabarinus*, *C. holoxanthus*, *C. malicorius*, and *C. sanguineus*) was explored by studying the minimal inhibitory concentrations (MIC) via a light-modified broth microdilution assay based on the recommended protocols of the European Committee on Antimicrobial Susceptibility Testing (EUCAST). The extracts were tested against *Candida albicans*, *Escherichia coli*, and *Staphylococcus aureus* under blue (λ = 428 and 478 nm, *H* = 30 J/cm^2^) and green light (λ = 528 nm, *H* = 30 J/cm^2^) irradiation. Three extracts showed significant photoantimicrobial effects at concentrations below 25 μg/mL. Targeted isolation of the major pigments from *C. sanguineus* led to the identification of two new potent photoantimicrobials, one of them (i.e., dermocybin) being active against *S. aureus* and *C. albicans* under green light irradiation [PhotoMIC^530^ = 39.5 μM (12.5 μg/mL) and 2.4 μM (0.75 μg/mL), respectively] and the other one (i.e., emodin) being in addition active against *E. coli* in a low micromolar range [PhotoMIC^428^ = 11.1 μM (3 μg/mL)]. Intriguingly, dermocybin was not (photo)cytotoxic against the three tested cell lines, adding an additional level of selectivity. Since both photoantimicrobials are not charged, this discovery shifts the paradigm of cationic photosensitizers.

Microbial resistances are inescapable and omnipresent not only in hospitals but also in centuries-old mummies, permafrost, and remote reaches of the ocean.^1^ According to the World Health Organization (WHO, 2021), antimicrobial resistance is one of the greatest global threats. Next to antimicrobial stewardship, developing new treatment strategies and antimicrobials is imperative to limit the dystopic predictions of 10 million deaths per year in 2050.^2^ In 2017, a list was published by the WHO, categorizing 12 microorganisms into three R&D priority classes (i.e., critical, high, and medium) for new antibiotics next to *Mycobacterium tuberculosis.*(3) In this list, five of the six nosocomial ESKAPE organisms^4^ are included (i.e., *Enterococcus faecium, Staphylococcus aureus*, *Acinetobacter baumannii*, *Pseudomonas aeruginosa*, and *Enterobacter* spp.). According to the committee establishing this catalog, future development should generally focus on antimicrobials active against tuberculosis and Gram-negative bacteria.^5^

A special type of antimicrobials, called photoantimicrobials, belongs to one of the emerging treatment strategies for microbial infections, i.e., antimicrobial photodynamic therapy (aPDT), which is also called photoantimicrobial chemotherapy (PACT) or photodynamic inhibition (PDI).^6−10^ The combination of light and a drug induces lethal signals in microorganisms, where well-established antimicrobials fail. Although the principle behind this was discovered by von Tappeiner and Oscar Raab 120 years ago,^11−13^ the potential of aPDT was barely realized before the manifestation of the current antimicrobial crisis.^14−17^ A pivotal role in the research of antimicrobial photodynamic chemotherapy was played by the group of Wainwright, who described the great potential of photodynamic antimicrobial therapy against human pathogenic drug-resistant strains.^18−20^ Intriguingly, resistances to photoantimicrobials have not yet been observed in the treated microorganisms.^9^ Thus, PACT is especially promising for treating local infections caused by multidrug-resistant microbes.^6,9,21^ For example, chlorin e6, a natural photosensitizer isolated from the alga chlorella (*Chlorella ellipsoidea*), is photoactive against 15 drug-resistant strains of *S. aureus.*(22)

Crucial parameters for such photosensitizers are the photoyield (i.e., the percentage of absorbed light that is transformed into the toxic reactive oxygen species singlet oxygen) and the absorbance spectra.^23^ In particular, for photoantimicrobials cellular uptake is essential,^24,25^ especially for Gram-negative bacteria, such as *Escherichia coli.*(26) The partial negative character of their outer membrane impedes the uptake of neutral or negatively charged photosensitizers.^27,28^ Thus, Gram-negative bacteria are relatively resistant to aPDT (or PACT) compared to Gram-positive bacteria, which take up photosensitizers more easily due to their porous peptidoglycan layers. Consequently, an established dogma of PACT says cationic photosensitizers are needed to effectively treat Gram-negative bacteria.^7,26^ One hypothesized mode of action is that cationic photosensitizers will more likely interact with the membrane and thus enable its lethal disruption by light irradiation. As a consequence of this accepted principle, most of the new photosensitizers against Gram-negative bacteria are characterized by at least one positive charge.^29−31^

As part of our ongoing efforts to investigate photosensitizers from mushrooms (i.e., from fruiting bodies of basidiomycetes),^32^ we were interested in exploring the potential of the colorful *Dermocybe* group against different microorganisms. Dermocybes are known for their brightly colored gills, which is due to their versatile (pre)anthraquinones, and were recognized as a subgenus of the species-rich genus *Cortinarius* belonging to the family Cortinariaceae. However, recent findings suggest that the family can be subdivided into ten individual genera.^33^ Dermocybes are characterized by a non-hygrophanous pileus and a non-bulbous stem next to their cortina or the residue of it on the stem. In 2010, Beattie et al.^34^ screened 117 collections of dermocyboid Cortinarius from Australia against *S. aureus* and *P. aeruginosa*, finding promising activities based on polyketides. Recently, we could show that photosensitizers are produced by representatives of related species.^35^*Cortinarius uliginosus,* as an example, produces the dimeric anthraquinone 7,7′-biphyscion, which was active in the nanomolar range under blue light irradiation against cells of several cancer cell lines.^36^ In another study, the extract of *C. rubrophyllus* showed promising photoactivated action against *S. aureus.*(37) However, the active anthraquinones were not isolated nor characterized. Nevertheless, from other sources, we know that the photoantimicrobial activity of natural anthraquinones is intriguing. For parietin, which can be isolated from the lichen *Xanthoria parietina*, a PhotoMIC of 56 μM (16 μg/mL) (*S. aureus*) was reported under interval irradiation [λ = 428 nm, *H* = 4.68 J/cm^2^, preincubation time (*t*_PI_) = 10 min].^38^ Aloe-emodin was i.a. isolated from the plants *Aloe vera* or *Rheum palmatum*; under irradiation a concentration of *c* = 10 μM (2.7 μg/mL) (λ = 435 ± 10 nm, *t*_PI_ = 2 h, *H* = 72 J/cm^2^) led to a total inhibition of the growth of the human pathogenic fungus *Trichophyton rubrum.*(39)

Here, we report on the photoantimicrobial screening of *C. malicorius* and related species, i.e., *Cortinarius cinnabarinus, C. sanguineus*, and *C. holoxanthus*, as well as the isolation and characterization of the photoantimicrobials from *C. sanguineus*.

## Results and Discussion

### Initial Photoantimicrobial Screening of Dermocyboid *Cortinarii*

Extracts of the four dermocyboid *Cortinarii* (Table S1, Figures S1–S4) were prepared with acidified acetone. After evaporating the solvent, the dried extracts were dissolved in DMSO and submitted to a primary photoactivity screening, consisting of (i) HPLC-DAD-MS profiling to characterize the pigmentation profile, (ii) the 9,9′-dimethylanthracene (DMA) assay to test the extracts’ ability to produce singlet oxygen in a cell-free environment (Figure S5), and (iii) a photoantimicrobial assay to explore any relevant photoactivity. As depicted in Figure S2, the pigment profile across the fruiting bodies of the four selected fungi varied intensely for all but *C. malicorius* and *C. sanguineus*, which are more closely related. The main pigments (at a detection wavelength of λ = 428 nm, Figure S6) were annotated by comparing the UV/vis and MS traces with in-house data (refer to Figure S7).

In detail, cinnalutein and fallacinol were annotated as the main orange and yellow pigments in *C. cinnabarinus*, flavomannin-6,6′-dimethyl ether (FDM) for *C. holoxanthus*, and emodin for *C. malicorius* and *C. sanguineus.* These observations are in agreement with the literature^40^ and our previous insights.^35^ In the next step, the extracts were submitted to the DMA assay,^41^ determining the relative singlet oxygen production as compared to berberine. The assay (Figure S5) is based on the formation of an endoperoxide and the subsequent quenching of the anthracene’s absorption properties. Additional controls are included to confirm singlet oxygen as reactive species, i.e., the quenching of the effect by l-ascorbic acid and an absorption scan before and after the irradiation cycle. A mixture of ethanol and DMSO was used for the extracts and the reference compound, respectively, to ensure comparability. The results revealed that under blue light irradiation, *C. cinnabarinus* is the most efficient producer of singlet oxygen (η_Δ,BL_ = 201%), followed by *C. sanguineus* (η_Δ,BL_ = 151%), *C. malicorius* (η_Δ,BL_ = 144%), and *C. holoxanthus* (η_Δ,BL_ = 64%). While the first three are potent producers of singlet oxygen, the extract of *C. holoxanthus* was less active. This might indicate (i) a weak singlet oxygen producer in the extract, (ii) a lower content, or (iii) the existence of a potent antioxidant, such as glutathione for example,^42^ in the extract.

A similar trend was observed under green light irradiation, though the difference between the yellow *C. holoxanthus* (η_Δ,GL_ = 33%) and the orange *C. malicorius* (η_Δ,GL_ = 48%), as well as *C. sanguineus* (η_Δ,GL_ = 58%), decreased. The latter can be explained by the lack of pigments absorbing green light as observed in the HPLC-DAD chromatogram detected at λ = 519 nm (Figure S3).

**Figure 1:**
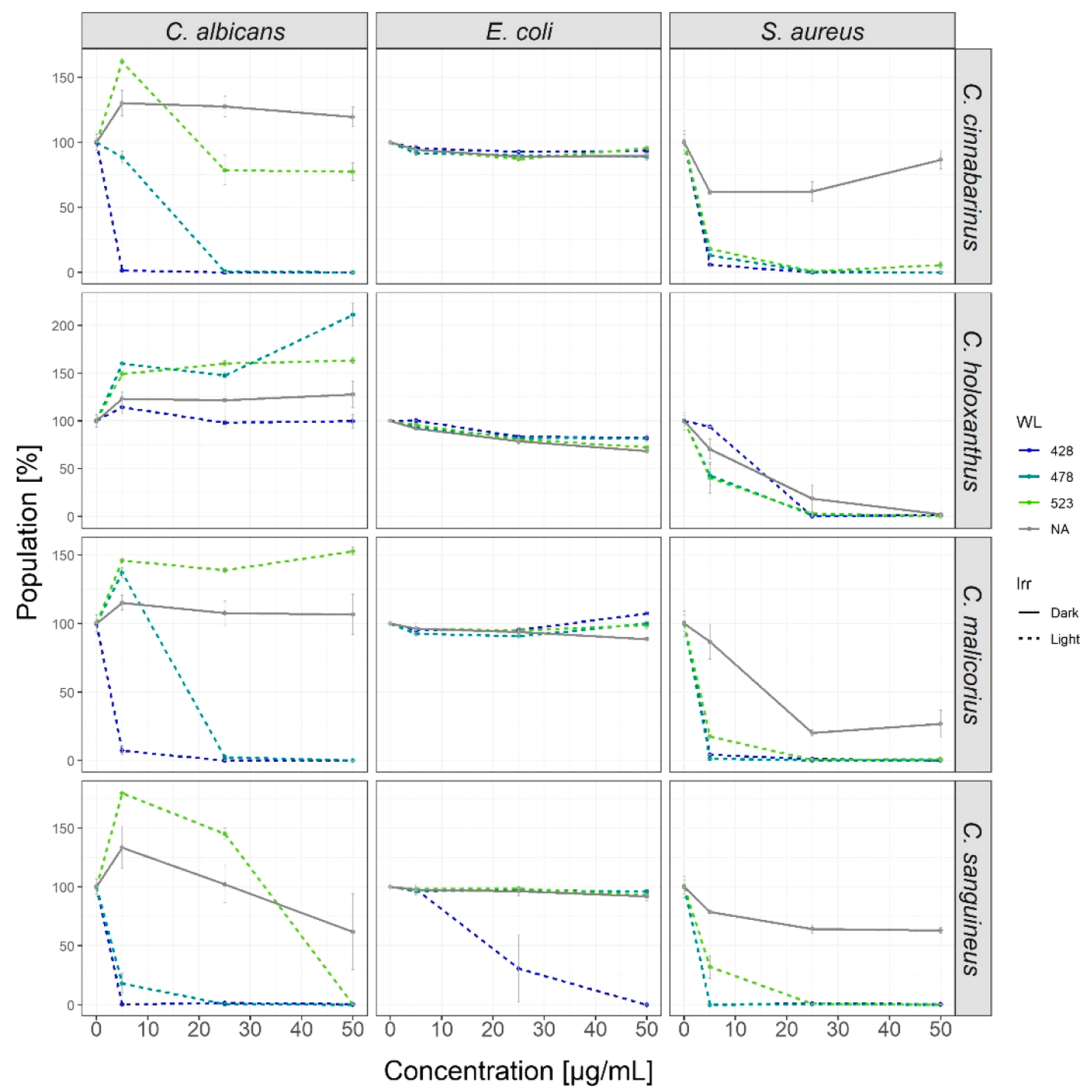
Dose–response curves of the extracts against the three tested microorganisms under irradiation (dotted lines, violet, blue, and green, all 30 J/cm^2^) as well as in the dark. WL = wavelength; Irr = irradiation condition.

The antimicrobial screening (Figure 1 and Figure S8) of the extracts revealed great variability across the four different species: While all extracts produced singlet oxygen, the antibacterial activity of the *C. holoxanthus* extract against *S. aureus* was independent of the irradiation parameters (i.e., blue light, green light, or darkness) and determined to be between *c* = 25 and 50 μg/mL. Thus, *C. holoxanthus* seems to contain at least one classic antimicrobial. One main component of the *C. holoxanthus* extract is FDM. There are no reports of studies exploring its antimicrobial effect in the literature, although the related fungal metabolite flavomannin A was studied in detail. In 2013, it was shown that the different atropisomers of flavomannin A have a MIC of approximately 16 μg/mL (29.3 μM) against *S. aureus* (ATCC 29213).^43^ Thus, the antimicrobial activity might be caused by flavomannin-6.6′-dimethyl ether. The other three extracts could be characterized by a wavelength-dependent photoantimicrobial effect against *C. albicans.* With photoenhanced minimum inhibitory concentrations (PhotoMIC) below or around 5 μg/mL, the extracts of *C. cinnabarinus*, *C. malicorius*, and *C. sanguineus* were highly active. This is, besides an initial observation regarding *C. malicorius*, which was reported previously by us,^37^ the first systematic proof of the photoantimicrobial potential of these mushrooms.

Of particular interest, however, was the observed activity of *C. sanguineus* against *E. coli*. An activity against Gram-negative bacteria is of importance, as four of the six ESKAPE organisms are Gram-negative bacteria (i.e., *Klebsiella pneumoniae*, *Acinetobacter baumannii*, *Pseudomonas aeruginosa*, and *Enterobacter* spp.), and thus, treating Gram-negative bacteria is one of the most critical therapeutic challenges today.^44,45^ The fact that the known pigments of these fruiting bodies are all uncharged, neutral aromatic compounds challenges the dogma that a positive charge is essential for photosensitizers to show any photoactivity against Gram-negative bacteria. To identify the active principle, the red extract of *C. sanguineus* was submitted to photoactivity-guided isolation.

### Targeted Isolation of Anthraquinones from *Cortinariussanguineus*

Extracts of different polarities [i.e., petroleum ether (PE), dichloromethane (DCM), and methanol (MeOH)] were prepared first to narrow the chemical nature of the photoantimicrobial principle of the bloodred webcap (*C. sanguineus*). Evaporation of the extraction solvents yielded extracts in the form of highly viscous liquids, which were redissolved in DMSO and subjected to an HPLC-DAD analysis as well as to the photoantimicrobial testing. The liquid-chromatographic analysis at λ = 468 nm (Figure S9) revealed that the DCM extract quantitatively contained the most anthraquinones. However, the variability of the enriched anthraquinones was more limited in the DCM extract than in the methanol extract. While the methanol extract consisted of monomeric and glycosylated anthraquinones, only the monomeric anthraquinones emodin (**4**) and dermocybin (**5**) were annotated in the DCM extract (Table S2).

**Figure 2:**
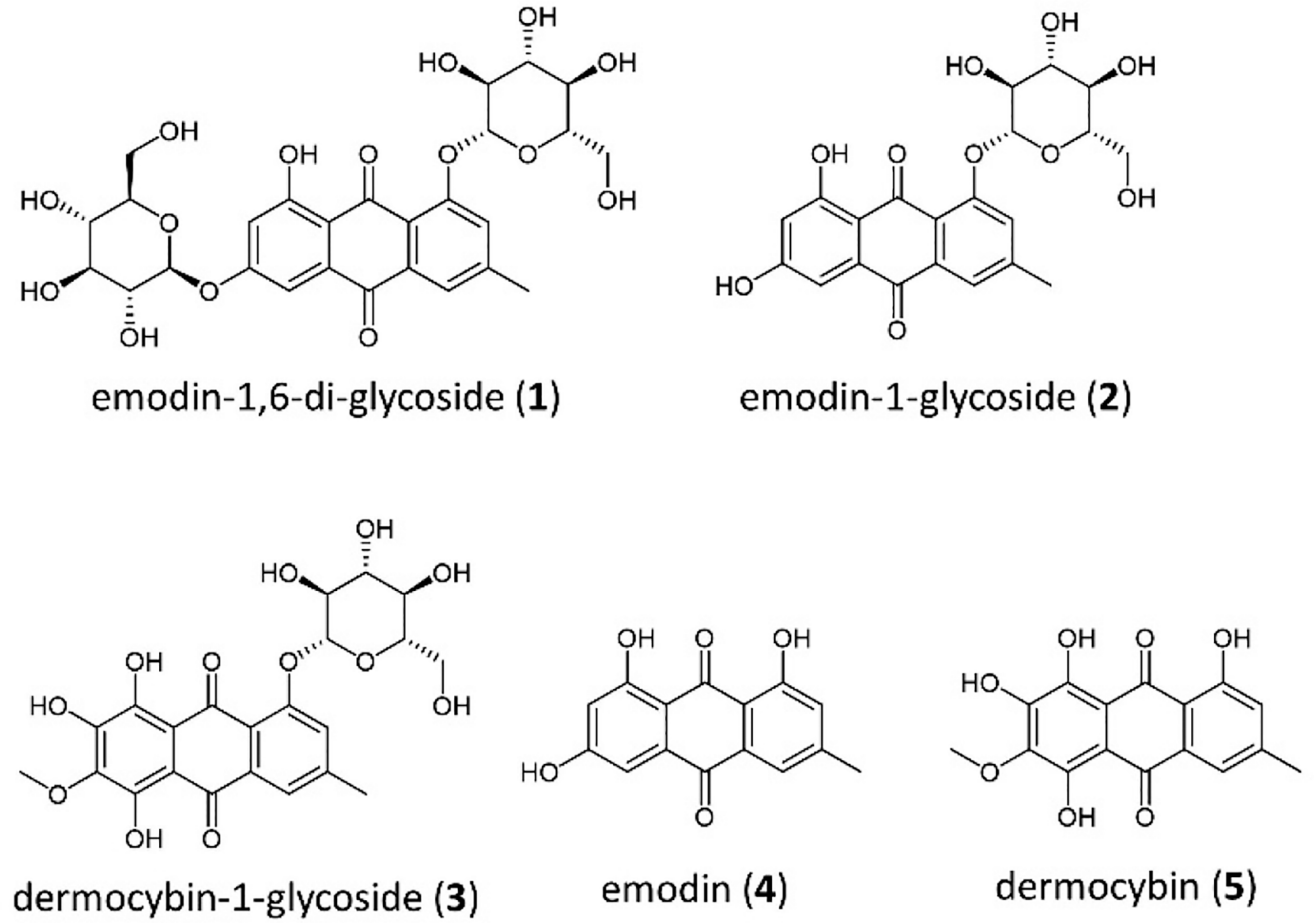
Isolated anthraquinones from *C*. *sanguineus*.

The photoantimicrobial assay (Figure S10) showed that the activity against *C. albicans* and *S. aureus* was retained, though the toxicity in the dark increased, at least for the apolar DCM extract. Nevertheless, the PhotoMIC values of all fractions were determined to be between *c* = 2 and 3 μg/mL, which demonstrates the photoantimicrobial potential of this fungal species. Against *E. coli*, the DCM extract showed a promising PhotoMIC of 3 μg/mL after irradiation at λ = 428 nm. By comparing the chromatograms of the extracts detected at λ = 428 and 468 nm, emodin was identified as the putative active principle, as the other compounds were less concentrated in the extract. However, for the activity against *S. aureus* and *C. albicans*, a clear hypothesis could not be drawn, as all three fractions showed activity. Thus, the two main components of the DCM extract and the glycosylated compounds of the polar region were isolated by different techniques (i.e., column chromatography, liquid–liquid extraction, and recrystallization) to test the photoantimicrobial potential of the individual compounds. In summary, five anthraquinones previously described^46,47^ were isolated from *C. sanguineus* (Figure 2). One of them, dermocybin-1-*O*-β-d-glucopyranoside (**3**), was, for the first time, characterized by means of IR spectroscopy (Figure S11), UV/vis spectroscopy (Figure S12), MS-spectrometry, and NMR spectroscopy (Figures S15–S20). In Table S2, the compiled NMR characterization of **3** and **5** is given.

### Photochemical and Photobiological Evaluation of Emodin (**4**) and Dermocybin (**5**)

The isolated monomeric anthraquinones of the most active fraction (DCM) and the glycosylated anthraquinones of the methanolic fraction, which are active against *S. aureus*, were photophysically examined. As listed in Table 1, emodin (**4**) was, in terms of the singlet oxygen photoyield, the most active anthraquinone of *C. sanguineus*, correlating with our insights from *C. rubrophyllus.*(35) In contrast, the bright pinkish dermocybin (**5**), holding two additional hydroxy groups at positions 5 and 7, was a poor photosensitizer. The two additional hydroxy groups induced a bathochromic shift of nearly Δλ = 100 nm. Glycosylation of one hydroxy group and thus the modification of the inductive effect did not affect the vis absorbance pattern or the singlet oxygen yield but decreased the molar extinction coefficient ε. In analogy, emodin (**4**) and its monoglycosylated derivative (**2**) were characterized by a similar absorbance pattern and a decrease in the absorbance coefficient. The singlet oxygen photoyield decreased by a factor of approximately two due to the glycosylation. Intriguingly, for **1**, a second glycoside induced an observable hypsochromic effect and a drastic epsilon reduction but no further decrease of the singlet oxygen quantum yield.

**Table 1:** Photophysical Characteristics of the Anthraquinone Metabolites Isolated from *C*. *sanguineus*^a^

compound	λ_max_ (ε) MeOH [nm (L mol^–1^ cm^–1^)]	λ_max_ (ε) ACN [nm (L mol^–1^ cm^–1^)]	φ_Δ_ (MeOD)	φ_Δ_ (ACN)
**1**	221 (4398), 264 (3551), 414 (1328)	220 (1680), 264 (1256), 282sh (1021), 411 (461)	0.09	0.02
**2**	212 (25970), 252 (19466), 287 (17096), 428 (6239)	216 (24888), 252 (17305), 267 (16990), 284 (19004), 416 (7313)	0.11*	0.02
**3**	215 (33720), 264 (29257), 485 (10742), 525sh (6869)	214 (28233), 264 (24783), 437 (8885), 363 (4960), 522sh (5085)	0.01	0.01
**4**	226 (16806), 287 (17167), 436 (10724)	222 (36083), 254 (16819), 266 (18235), 288 (19063), 433 (11755)	0.28*	0.49
**5**	219 (31518), 262 (28581), 277 (23518), 459sh (12966), 484 (15450), 517 (11202)	218 (22927), 261 (22106), 275sh (17836), 303 (8052), 459sh (9911), 483 (12099), 514sh (8106)	0.04	0.01

a Perinaphthenone is used as a reference with reported Φ_Δ_(^1^O_2_) = 0.98 ± 0.07 in air-saturated ACN.^48^ [Ru(bpy_3_)]Cl_2_ is used as a validation compound with reported Φ_Δ_(^1^O_2_) = 0.57 ± 0.06 in air-saturated ACN.^49^ All the samples were dissolved in *V* = 3 mL of ACN or MeOD and transferred into a macro fluorescence cuvette (Firefly; light path: 1 cm × 1 cm). The ^1^O_2_ phosphorescence was measured at 298 K using a 450 nm fiber-coupled laser set to 80 mW. The NIR emission spectra were acquired within 20 s. *Values adapted from ref (35).

As glycosylation is accepted as an energy storage principle in Nature, the enrichment of glycosylated anthraquinones in the fruiting bodies might have resulted from this triadic benefit: (i) The photosensitizer is deactivated as compared to the aglycon and thus less harmful for the fungi itself; (ii) it is a convenient energy storage, and the water solubility is enhanced, combined with (iii) a sophisticated defense function being activated via the metabolism of the fungivore. Next, the PhotoMICs of the isolated compounds were determined according to a modified EUCAST protocol.^50^ For each compound, a series of at least ten different concentrations were tested in biological triplicates against *C. albicans*, *E. coli*, and *S. aureus*. An identical treated plate was kept in the dark to confirm that the observed effects were indeed caused by the synergistic action between light and the fungal metabolites. Table 2 summarizes the obtained results. While all glycosides were inactive, the monomeric anthraquinones dermocybin (**5**) and emodin (**4**) were of particular interest.

**Table 2:** PhotoMIC Values and MIC Values of the Isolated Anthraquinones from the Genus *Cortinarius sanguineus*^a^

PS	irradiation	*Candida albicans*	*Escherichia coli*	*Staphylococcus aureus*
**1**	428 nm (30 J/cm^2^)	>84 μM (50.00 μg/mL)	>84 μM (50.00 μg/mL)	>84 μM (50.00 μg/mL)
	478 nm (30 J/cm^2^)			
	530 nm (30 J/cm^2^)			
	dark			
**2**	428 nm (30 J/cm^2^)	>115 μM (50.00 μg/mL)	>115 μM (50.00 μg/mL)	>115 μM (50.00 μg/mL)
	478 nm (30 J/cm^2^)			
	530 nm (30 J/cm^2^)			
	dark			
**3**	428 nm (30 J/cm^2^)	>105 μM (50.00 μg/mL)	>105 μM (50.00 μg/mL)	>105 μM (50.00 μg/mL)
	478 nm (30 J/cm^2^)			
	530 nm (30 J/cm^2^)			
	dark			
**4**	428 nm (30 J/cm^2^)	0.37 μM (0.10 μg/mL)	11.1 μM (3.00 μg/mL)	1.85 μM (0.50 μg/mL)
	478 nm (30 J/cm^2^)	2.78 μM (0.75 μg/mL)	>92 μM (25.00 μg/mL)	1.85 μM (0.50 μg/mL)
	530 nm (30 J/cm^2^)	46 μM (12.50 μg/mL)	>92 μM (25.00 μg/mL)	11.1 μM (3.00 μg/mL)
	dark	>92 μM (25.00 μg/mL)	>92 μM (25.00 μg/mL)	>92 μM (25.00 μg/mL)
**5**	428 nm (30 J/cm^2^)	39.5 μM (12.50 μg/mL)	>79 μM (25.00 μg/mL)	39.5 μM (12.50 μg/mL)
	478 nm (30 J/cm^2^)	19.7 μM (6.25 μg/mL)		4.74 μM (1.50 μg/mL)
	530 nm (30 J/cm^2^)	39.5 μM (12.50 μg/mL)		2.37 μM (0.75 μg/mL)
	dark	>79 μM (25.00 μg/mL)		>79 μM (25.00 μg/mL)
amphotericin	dark	0.22 μM (0.20 μg/mL)		
chloramphenicol	dark		6.1 μM (2.00 μg/mL)	
erythromycin	dark			1.4 μM (1.00 μg/mL)

a Preincubation time was set to *t* = 60 min.

Dermocybin (**5**) was the most active compound under green light irradiation. A concentration as low as *c* = 2.37 μM or 0.75 μg/mL was enough to impede the growth of *S. aureus*. The effect was, however, not solely induced by light irradiation, as **5** caused a decrease of 50–70% in the dark. Nevertheless, under the chosen concentrations the dark toxicity of **5** was not efficient enough to prompt a complete growth inhibition; thus, a MIC value could not be determined. Additionally, since **5** showed only limited ability to transform the absorbed photoenergy into singlet oxygen [φ_Δ_(MeOD) = 4%], it can be assumed that it acts as a PDT type 1 rather than type 2 photosensitizer and thus that the production of superoxide anion is involved. A similar phenomenon was reported for the antibacterial effect of, for example, anthraquinone derivatives from the plant *Heterophyllaea pustulata.*(51) Besides showing photoantimicrobial properties, the 5,5′-bisoranjidiol, among other derivatives, also induced a bacterial static effect under dark conditions. The antimicrobial effect was correlated to an increased superoxide anion production, which occurred in the dark and was enhanced under actinic irradiation. Furthermore, Comini and co-workers investigated the production of singlet oxygen. It was shown that the ^1^O_2_ levels were significantly increased under irradiation, as compared to the dark control, explaining the strong bactericidal effect.^51^ For other anthraquinones from *H. pustulata*, i.e., rubiadin and rubiadi-1-methyl ether, superoxide anion was supposed to be the active ROS species in *Candida tropicalis* biofilms^52^ and supports the assumption that **5**’s mode-of-action might be PDT type 1 mediated.

The possibility to induce the photoeffect by green light irradiation is of special interest due to the deeper tissue penetration compared to blue light irradiation. Furthermore, an *in vitro* cytotoxicity assay employing cells of mammal cell lines (Table 3) revealed that **5** is only moderately toxic; weak toxicity (EC_50_ = 16.2 μM) was observed under green light irradiation against cells of the lung cancer cell line A549, while it was nontoxic in the dark. **5** was not cytotoxic against the stomach cell line AGS, neither in the dark nor under irradiation. Nevertheless, the light-induced effect, in general, is not as strong as the one of emodin (**4**), which can be rationalized by the low singlet oxygen photoyield, and showed in the tested concentration range no dark toxicity. Dermocybin (**5**) might act as a mixed type PS (i.e., PDT type I and type II); however, further studies are needed to explore its mode-of-action in detail.

**Table 3:** EC_50_ Values (μM) as Determined by the (Photo)cytotoxicity Assay of Emodin-1,6-di-*O*-β-d-glucopyranoside (**1**), Emodin-1-*O*-β-d-glucopyranoside (**2**), Dermocybin-1-*O*-β-d-glucopyranoside (**3**), Emodin (**4**), and Dermocybin (**5**)^a^

	A549 (BL, 468 nm)		A549 (dark)		P.I.	AGS (BL, 468 nm)		AGS (dark)	P.I.	T24 (BL, 468 nm)		T24 (dark)	P.I.
**1**	>25.0		>25.0			>25.0		>25.0		>25.0		>25.0	
**2**^b^	>25.0		>25.0			>25.0		>25.0		>25.0		>25.0	
**3**	>25.0		>25.0			>25.0		>25.0		>25.0		>25.0	
**4**^b^	2.9	1.1	19	5.3	7	1.5	0.9	>25.0	>16	1.7	0.6	>25.0	>15
		0.8		4.1			0.6				0.4		
**5**	>25.0		>25.0			>25.0		>25.0		>25.0		25	

	A549 (GL, 519 nm)		A549 (dark)	P.I.	AGS (GL, 519 nm)	AGS (dark)	P.I.	T24 (GL, 519 nm)		T24 (dark)		P.I.
**3**	>25.0		>25.0		>25.0	>25.0		>25.0		>25.0		
**5**	16.2	4.1	>25.0	>1.5	>25.0	>25.0		11.6	4.4	24.7	13	2.1
		3.3							3.2		9	

a The dark cytotoxicity as well as the blue/green light-dependent cytotoxicity (blue light irradiation: λ = 468 ± 27 nm, 9.3 J/cm^2^, green light irradiation: λ = 519 ± 33 nm, 30.0 J/cm^2^) was evaluated. EC_50_ values in combination with their 95% confidence intervals are given in μM. The photoindex, calculated as the ratio of cells killed in the dark to cells killed under irradiation, is given as well.

b Adopted from ref (35).

In contrast to dermocybin (**5**), **4** is active against all three microorganisms tested. *C. albicans* and *S. aureus* were killed by **4** at doses as low as *c* = 0.37 and 1.85 μM, respectively, when irradiated close to its absorbance maximum. Emodin (**4**) is even more active as a derivative [i.e., 3-(bis(3-(dimethylamino)propyl)amino)-7-(di-*n*-propylamino)phenothiazinium iodide] of the established photosensitizer methylene blue, which is characterized by a PhotoMIC of 3.13 μM (1.00 μg/mL) against *C. albicans.*(29) Also, compared to the related natural product aloe-emodin (log 6 reduction achieved with *c* = 10 μM, *t*_PI_ = 30 min, λ = 400–780 nm, *H* = 24.8 J/cm^2^ ^39^), **4** seems to be more promising. Most strikingly, **4** was active against *E. coli*, contrasting the current state of knowledge (i.e., the requirement of a cationic character for a photosensitizer to be active against Gram-negative bacteria).^7,26^ It seems that the neutral and flat anthraquinone with a logP of 3.641 can penetrate the outer membrane and is thus able to kill the bacteria when light-triggered. Though its photophysical properties and especially its ability to produce singlet oxygen have been known since 1992,^53^ the exploration of its photoantimicrobial effect is reported here for the first time. Furthermore, **4**’s high activity is of special interest: For other photoantimicrobials, for example for the related hypericin, an optimal concentration against *E. coli* of *c* = 36 μM (*c* = 18 μg/mL, *t*_PI_ = 68 min, λ = 590 nm, *H* = 5.9 J/cm^2^) was found.^54^

## Conclusion

It is shown that extracts of the colorful dermocyboid *Cortinarii* are characterized by promising photoantimicrobial activities. While the moderate antimicrobial potential of mushrooms under dark conditions was known already, the additional effect of light is reported here in detail for the first time. Under light irradiation, the antimicrobial effect is enhanced, reaching concentrations as low as 0.37 and 2.27 μM for the two monomeric anthraquinones emodin (**4**) and dermocybin (**5**). The observed action of **5** under green light irradiation against *S. aureus* is propitious due to its selective character; an *in vitro* cytotoxicity test employing cells of an immortal human stomach cell line (AGS) showed no (photo)cytotoxic effect. Most intriguingly, however, the abundant natural compound emodin (**4**) was highly active against *C. albicans* and *S. aureus* under blue light irradiation [PhotoMIC^428^*c* = 0.1 μg/mL (0.37 μM) and 0.5 μg/mL (1.85 μM)] and was active against *E. coli* [PhotoMIC^428^ = 3 μg/mL (11 μM)]. While emodin was reported to occur in many plants, insects, lichens, and fungi, this is the first time its promising photoantimicrobial effect has been revealed. A systematic exploration of the many known natural and synthetic anthraquinones might even lead to more promising photoantimicrobial compounds.

## Experimental Section

### General Experimental Procedures

Solvents were purchased from VWR International (Vienna, Austria) if not stated otherwise. Acetone was distilled prior to use. Solvents for HPLC experiments had pro analysis (p.a.) quality and were obtained from Merck (Merck KGaA, Darmstadt, Germany). Ultrapure water was obtained with the Sartorius Arium 611 UV purification system (Sartorius AG, Göttingen, Germany). Curcumin, dimethyl sulfoxide (DMSO), lysogeny broth (LB) agar, and RPMI1640 medium were purchased from Merck KGaA (Darmstadt, Germany). Potato dextrose agar (PDA) and Mueller Hinton Broth (MHB) were purchased from VWR International (Vienna, Austria). The 96-well plates (flat bottom) were purchased from Sarstedt (Nümbrecht, Germany).

Desiccation of the fruiting bodies (FB) was done with a dehydrator from Stöckli (A. & J. Stöckli AG, Switzerland) operated at a temperature of 40 °C. The Bosch MKM 6003 coffee grinder (Stuttgart, Germany) was used for grinding. Scales from KERN ALS 220-4 (KERN & SOHN GmbH, Balingen-Frommern, Germany) and Sartorius Cubis-series (Sartorius AG, Göttingen, Germany) were employed. The Sonorex RK 106 ultrasonic bath (BANDELIN Electronic GmbH & Co. KG, Berlin, Germany) was utilized, as well as the Vortex-Genie 2 mixer (Scientific Industries, Inc., Bohemia, NY, USA). For centrifugation, an Eppendorf 5804R centrifuge with an F-45-30-11-30 place-fixed angle rotor (Hamburg, Germany) was used.

Pipetting was done with pipettes and tips from Eppendorf AG (Hamburg, Germany) and STARLAB International GmbH (Hamburg, Germany). Reagent reservoirs were obtained from Thermo Fischer Scientific (Waltham, MA, USA).

HPLC measurements were carried out with the Agilent Technologies 1260 Infinity II modular system (Agilent Technologies, Inc., Santa Clara, CA, USA) with a quaternary pump, vial sampler, column thermostat, diode-array detector, and mass spectrometer. Moreover, the Agilent Technologies 1200 Series HPLC system with a binary pump, autosampler, column thermostat, and diode-array detector was used. For all HPLC measurements, a Synergi 4 μm MAX-RP 80 Å 150 mm × 4.60 mm column was used. HPLC-DAD-ESI-MS analysis was carried out with the Agilent Technologies 1260 Infinity II modular system equipped with a quaternary pump, vial sampler, column thermostat, diode-array detector, and an ion trap mass spectrometer (amaZon, Bruker, Bremen, Germany). The U-2001 spectrophotometer for adjusting the McFarland standard was purchased from Hitachi (Chiyoda, Japan).

For measurement of the 96-well plates, a Tecan Sunrise remote plate reader (Tecan, Männedorf, Switzerland) was used. The adjustment of pH values was carried out with the Mettler Toledo SevenMulti pH-meter (Mettler-Toledo GmbH, Vienna, Austria).

Homogenous irradiation of the 96-well plates was achieved by utilizing either the Agilent E3611A DC power adaptor power supply (Agilent Technologies, Inc., Santa Clara, CA, USA) in combination with an LED panel [λ = 468 ± 27 nm (*E*_m_ = 20.6 mW cm^–2^) or λ = 519 ± 33 nm (*E*_m_ = 22.3 mW cm^–2^) (University Leiden, published in Hopkins et al.,^55^ characterized in Siewert et al.^56^)] or the SciLED setup [λ = 478 ± 22 nm (*E*_m_ = 8.7 mW cm^–2^) or λ = 523 ± 33 nm (*E*_m_ = 6.0 mW cm^–2^) published by Fiala and Schöbel et al.].^50^

### Fungal Biomaterial and Sample Preparation

The fruiting bodies of *C. cinnabarinus*, *C. holoxanthus*, *C. malicorius*, and *C. sanguineus* were collected in Tyrol (Austria) and Abruzzo (Italy). Table S1 contains detailed information and GenBank numbers. A reliable taxonomic classification was achieved by combining macroscopic and microscopic techniques as well as rDNA ITS sequence analysis. After collection and identification, the fresh fruiting bodies were immediately frozen and stored in a freezer at −18 °C or, in the case of *C. cinnabarinus*, dried in a dehydrator at *T* = 45 °C. Before extraction, the fruiting bodies were freeze-dried, finely ground with mortar and pestle, and stored in paper bags at room temperature until further use (*T* = 23.0 °C, humidity = 20 ± 10%).

### Preparation of Fungal Extracts

The biomaterials were milled and sieved utilizing a mesh with a size of 400 μm. The extraction process was performed under light exclusion at room temperature. The powdered materials (*m* = 2.00 g) were extracted with acidified acetone (*V* = 20 mL, 0.1% v/v 2 N HCl) in an ultrasonic bath (*t* = 10 min). After centrifugation (*t* = 10 min, *T* = 4 °C, RCF = 21000*g*), acetone was decanted and filtered through cotton wool. The procedure was repeated twice with smaller volumes of acidified acetone (*V* = 5 mL). The combined supernatants were evaporated and stored in brown glass vials at room temperature (see Table 1 for yields).

### Targeted Isolation of *C. sanguineus*

After drying and milling of the *C. sanguineus* fruiting bodies, the biomaterial (*m* = 34.8 g) was extracted successively with petroleum ether (*V* = 500 mL, *n* = 4), dichloromethane (*V* = 500 mL, *n* = 6), methanol (*V* = 500 mL, *n* = 10), and water (*V* = 500 mL, *n* = 1) by using ultrasonication (5 min per extraction step) at room temperature (*T* = 22.5 °C). Following each extraction step, the extracts were filtered, and the respective filtrates were combined. After solvent evaporation, the extracts were subjected to freeze-drying for removal of any residual solvent. The extraction process yielded η = 577.6 mg (1.7% w/w) of petroleum ether extract, η = 979.6 mg (2.8% w/w) of dichloromethane extract, η = 12665.8 mg (36.4% w/w) of methanol extract, and η = 2913.0 mg (8.4% w/w) of water extract.

#### Isolation of Emodin-1,6-di-*O*-β-d-glucopyranoside (**1**)

An aliquot of the *C. sanguineus* methanol extract (*m* = 3.27 g) was dissolved in ultrapure water (*V* = 350 mL), transferred into a separatory funnel, and extracted successively with diethyl ether (Et_2_O, *V* = 400 mL, *n* = 7), ethyl acetate (EtOAc, *V* = 300 mL, *n* = 8), and water-saturated *n*-butanol (BuOH, *V* = 200 mL, *n* = 4) by liquid–liquid extraction. The respective fractions and the residual aqueous phase (H_2_O) were dried by vacuum rotary evaporation at 40 °C. Any solvent residues were removed by freeze-drying. The yields of the fractions were as follows: *m*_Et2O_ = 198.5 mg (6.1% w/w), *m*_EtOAc_ = 175.8 mg (5.4% w/w), *m*_BuOH_ = 461.3 mg (14.1% w/w), and *m*_H2O_ = 2206.0 mg (67.5% w/w). Subsequently, an aliquot of the *n*-butanol fraction (*m* = 300.2 mg) was dissolved in ultrapure water (*V* = 200 mL) and subjected again to a successive liquid–liquid extraction with dichloromethane (BuL1, *V* = 200 mL, *n* = 3) and ethyl acetate (BuL2, *V* = 200 mL, *n* = 9). Thereafter, the aqueous phase was acidified with glacial acetic acid until the color changed from red to orange and extracted with ethyl acetate (BuL3, *V* = 200 mL, *n* = 6). The fractions were dried as described above, and the yields determined: *m*_BuL1_ = 5.8 mg (1.9% w/w), *m*_BuL2_ = 42.5 mg (14.2% w/w), and *m*_BuL3_ = 90.7 mg (30.2% w/w).

The residual aqueous phase was submitted to RP-18 solid-phase extraction (RP-18 SPE, stationary phase: LiChroprep RP-18 (0.040–0.063 mm), ⦶ = 15 mm, l = 25 mm). After activation of the column with methanol, it was equilibrated with water and loaded with the aqueous phase. The loaded column (trapped metabolites were visible as a yellow band) was washed with water (*V* = 20 mL), and then elution was performed with methanol (*V* ∼ 10 mL). The fraction obtained (BuL4) was dried under air flow and yielded a mass of 27.1 mg (7.0% w/w). BuL4 (*m* = 14.3 mg) was purified by preparative thin-layer chromatography (TLC/stationary phase: precoated TLC sheets, 10 × 20 cm, silica gel 60 F254 0.20 mm layer) with toluene–acetone–formic acid–acetic acid (35:40:12.5:12.5) as mobile phase. For this purpose, BuL4 was dissolved in methanol (*V* ∼ 2 mL) and loaded onto seven TLC plates. After separate development (separation distance ∼8 cm), the yellow band with an *R*_*f*_ value of 0.2 was removed from each plate, suspended via sonication in water, and purified via RP-18 SPE as described above. Elution of **1** (4.2 mg) was performed with acetonitrile.

Emodin-1,6-di-*O*-β-d-glucopyranoside (**1**) was obtained as a yellow solid (η = 4.2 mg, 0.012% w/w). Mp: no clear mp observed (decomposition >250 °C); [α]_D_^25^ = −56 (*c* = 0.10 mg/mL, MeOH); UV–vis (MeOH) λ_max_ (ε) = 221 (4398), 264 (3551), 414 nm (1328 mol^–1^ dm^3^ cm^–1^); IR (ART) ν̃ = 3344 (w), 2924 (w), 1629 (w), 1262 (w), 1068 (w) cm^–1^; ^1^H NMR (600 MHz, D_2_O, 25 °C) δ = 7.19 (s, 1H, C_ar_*H*-2), 7.13 (s, 1H, C_ar_*H*-4), 6.84 (d, *J* = 2.4 Hz, 1H, C_ar_*H*-5), 6.62 (d, *J* = 2.4 Hz, 1H, C_ar_*H*-7), 5.14 (d, *J* = 7.6 Hz, 1H, C*H*-1″), 4.99 (d, *J* = 6.8 Hz, 1H, C*H*-1′), 4.12–4.04 (m, 2H, C*H*_a_-6′ + C*H*_a_-6″), 3.97–3.88 (m, 2H, C*H*_b_-6′ + C*H*_b_-6″), 3.82–3.62 (m, 8H, glycosidic H), 2.31 (s, 3H, C*H*_3_-3) ppm; MS (ESI, negative mode 4.5 kV) *m*/*z* (%) 629.0 (100), 656.0 (85), 431.0 (86), 593.0 (66) [M – H]^−^; *R*_*f*_ = 0.20 (stationary phase: SiO_2_, mobile phase: toluene–acetone–formic acid–acetic acid = 35:40:12.5:12.5).

#### Isolation of Emodin-1-*O*-β-d-glucopyranoside (**2**) and Dermocybin-1-*O*-β-d-glucopyranoside (**3**)

An aliquot of the methanol extract (*m* = 1475.9 mg) was dissolved in ultrapure water (*V* = 150 mL), transferred into a separatory funnel, and extracted successively with diethyl ether (*V* = 100 mL, *n* = 8) and ethyl acetate (*V* = 150 mL, *n* = 5). The ethyl acetate extracts were combined and dried using vacuum rotary evaporation at 40 °C and yielded 94.6 mg (6.4% w/w) of the fraction called M1. The aqueous phase was then acidified with 20 mL of glacial acetic acid. The acidified phase was extracted once with 100 mL of ethyl acetate, and the mixture was set aside. Subsequently, extraction was carried out with ethyl acetate (*V* = 100 mL, *n* = 4). The extracts were combined and evaporated to dryness to finally obtain 430.2 mg of fraction M2. M1 (*m* = 73.2 mg) was further purified via repeated (*n* = 2) recrystallization from 60% ethanol. Briefly, the aliquot was suspended, heated in a water bath (*T* = 100 °C) until fully dissolved, and stored in a refrigerator (*T* = 8 °C) for several hours. The crystallized compound was then centrifuged, washed with water, and dried under an air flow. In this way, 4.7 mg of **2** were obtained. Purification of M2 by recrystallization from 60% ethanol as described above gave 7.7 mg of **3**.

Emodin-1-*O*-β-d-glucopyranoside (**2**) [CAS: 38840-23-2] was isolated as an orange solid (η = 4.7 mg, 0.014% w/w). Identification was achieved via comparison of compound-specific properties with reference data.^35^ Mp: 220–228 °C (239–241 °C,^57^ 210–211 °C^46^); UV–vis (MeOH) λ_max_ (ε) = 212 (25970), 252 (19466), 287 (17096), 428 nm (6239 mol^–1^ dm^3^ cm^–1^); IR (ART) = ν̃ = 3215 (w), 2921 (w), 1619 (w), 1594 (w), 1254 (w), 1176 (w), 1057 (w), 1023 (m), 882 (m), 719 (w), 521 (w), 420 (w) cm^–1^; ^1^H NMR (600 MHz, CD_3_OD, 25 °C) δ = 8.51 (s, 1H, C_ar_O*H*-6), 7.82 (s, 1H, C_ar_*H*-4), 7.63 (s, 1H, C_ar_*H*-2), 7.14 (s, 1H, C_ar_*H*-5), 6.55 (s, 1H, C_ar_*H*-7), 5.02 (d, *J* = 7.6 Hz, 1H, C*H*-1′), 3.97 (dd, *J* = 12.1, 2.3 Hz, 1H, C*H*_a_-6′), 3.74 (dd, *J* = 12.1, 6.3 Hz, 1H, C*H*_b_-6′), 3.70–3.64 (dd, *J* = 9.3, 7.6 Hz, 1H, C*H*-2′), 3.58–3.52 (m, 2H, C*H*-3′ and C*H*-5′), 3.46–3.42 (dd, *J* = 9.3, 9.3 Hz, 1H, C*H*-4′), 2.50 (s, 3H, C*H*_3_-3) ppm; MS (ESI, negative mode 4.5 kV) *m*/*z* (%) 431.7 (100) [M – H]^−^; *R*_*f*_ = 0.65 (stationary phase: SiO_2_, mobile phase: toluene–acetone–formic acid–acetic acid = 35:40:12.5:12.5).

Dermocybin-1-*O*-β-d-glucopyranoside (**3**) was obtained as a red solid (η = 7.7 mg, 0.022% w/w) with minor impurities of **2**. Mp: 232–234 °C (228–230 °C^46^); [α]_D_^20^ = −88 (*c* = 0.14 mg/mL, MeOH); UV–vis (MeOH) λ_max_ (ε) = 215 (33720), 264 (29257), 301 (shoulder/14554), 485 (10781), 525 nm (shoulder/6464 mol^–1^ dm^3^ cm^–1^); IR (ART) = ***ν̃*** = 3413 (w), 2917 (w), 1588 (w), 1471 (w), 1408 (w), 1299 (w), 1035 (w), 634 (w), 550 (w), cm^–1^; ^1^H NMR (600 MHz, CD_3_OD, 25 °C) δ 7.90 (s, 1H, C_ar_*H*-4), 7.59 (s, 1H, C_ar_*H*-2), 5.06 (d, *J* = 7.7 Hz, 1H, C*H*-1′), 4.02 (s, 3H, OC*H*_3_-6), 3.99 (dd, J = 12.1, 2.3 Hz, 1H, CH_a_-6′), 3.77 (dd, J = 12.1, 6.2 Hz, 1H, CH_b_-6′), 3.72–3.65 (m, 1H, C*H*-2′), 3.61–3.54 (m, 2H, C*H*-3′ and C*H*-5′), 3.49–3.44 (m, 1H, C*H*-4′), 2.53 (s, 3H, C*H*_3_-3) ppm; MS (ESI, negative mode 4.5 kV) *m*/*z* (%) 477.1 (100) [M – H]^−^; *R*_*f*_ = 0.55 (stationary phase: SiO_2_, mobile phase: toluene–acetone–formic acid–acetic acid = 35:40:12.5:12.5).

#### Isolation of Emodin (**4**)

An aliquot (*m* = 500.1 mg) of the dichloromethane extract was subjected to acetylated polyamide column chromatography (⦶ = 2.5 cm, l = 45 cm). First, isocratic elution was performed with petroleum ether (*V* = 200 mL), followed by elution with petroleum ether–toluene (8:2 v/v, *V* = 100 mL, D1), toluene (*V* = 700 mL), toluene–chloroform (9:1 v/v, *V* = 300 mL), toluene–chloroform (7:3 v/v, *V* = 200 mL), and last chloroform (*V*_CHCl3|1_ = 450 mL, *V*_CHCl3|2_ = 300 mL). Concentration of the second chloroform eluate (i.e., *V*_CHCl3|2_: approximately 50% of the starting volume left) via rotary evaporation *in vacuo* resulted in the precipitation of orange crystals. The precipitate was filtered off and dried to obtain 79.7 mg of **4**.

Emodin (**4**) [CAS: 518-82-1] was isolated as an orange solid (η = 79.7 mg, 0.23% w/w). Mp: 260 °C (253–254 °C^58^); UV–vis (MeOH) λ_max_ (ε) = 226 (16806), 287 (17167), 436 nm (10724 mol^–1^ dm^3^ cm^–1^); ^1^H NMR (600 MHz, DMSO-d6, 25 °C) δ = 12.09 (s, 1H, C_ar_-O*H*-8), 12.02 (s, 1H, C_ar_-O*H*-1), 11.34 (brs, 1H, C_ar_-O*H*-6), 7.51 (d, *J* = 1.6 Hz, 1H, C_ar_*H*-4), 7.18 (d, *J* = 1.8 Hz, 1H, C_ar_*H*-2), 7.13 (d, *J* = 2.4 Hz, 1H, C_ar_*H*-5), 6.60 (d, *J* = 2.4 Hz, 1H, C_ar_*H*-7), 2.47 ppm (s, 3H, C*H*_3_-3); MS (ESI, negative mode 4.5 kV) *m*/*z* (%) 269.0 (100) [M – H]^–^; *R*_*f*_ = 0.65 (stationary phase: SiO_2_; mobile phase: toluene–ethyl acetate–formic acid–acetic acid (70:20:5:5)).

#### Isolation of Dermocybin (**5**)

Aliquots of the water extract (*m* = 1636.6 mg) and the water fraction (*m* = 1066.8 mg) were combined, dissolved in ultrapure water, transferred into a separatory funnel, and extracted successively with diethyl ether (W1, *V* = 250 mL, *n* = 3) and ethyl acetate (W2, *V* = 300 mL, *n* = 4). Then, the aqueous phase was acidified with glacial acetic acid (*V* = 30 mL). The acidified phase was extracted five times with ethyl acetate (*V* = 300 mL), with the first two extracts being combined with fraction W3 and the last three with fraction W4. Solvents were removed using vacuum rotary evaporation at 40 °C and freeze-drying. The resulting fractions gave the following yields: *m*_W1_ = 54.4 mg (2.0% w/w), *m*_W2_ = 10.1 mg (0.4% w/w), *m*_W3_ = 7.5 mg (0.3% w/w), and *m*_W4_ = 181.8 mg (6.7% w/w). Fraction W1 (*m* = 21.0 mg) was dissolved in a mixture containing equal volumes of diethyl ether and dichloromethane (*V* = 50 mL), transferred into a separatory funnel, and extracted with a saturated Na_2_HPO_4_ solution (*V* = 50 mL, *n* = 3). The resulting fractions were combined, acidified with concentrated hydrochloric acid (q.s. for color change from purple to yellow), and extracted twice with 50 mL of diethyl ether–dichloromethane (1:1 v/v). The combined fractions were then evaporated to dryness and yielded 9.3 mg of crude dermocybin (W1.1). W.1.1 was further purified by suspending the dried fraction in a small volume of acetone (q.s. to 50% dissolution) and adding a saturated Na_2_HPO_4_ solution until completely dissolved. Then the solution was diluted with six times the amount of ultrapure water and extracted with diethyl ether (*V* = 100 mL, *n* = 2). Thereafter, the aqueous phase was acidified with concentrated hydrochloric acid (qs until color change from red to yellow) and extracted once with diethyl ether (W1.2, *V* = 100 mL). After drying as described above, fraction W1.2 yielded 6.3 mg of **5**.

Dermocybin (**5**) [CAS: 7229-69-8] was obtained as a red solid (η = 6.3 mg, 0.018% w/w). Mp: 220–222 °C (228–229 °C^58^); UV–vis (MeOH) λ_max_ (ε) = 219 (31518), 262 (28581), 277 (shoulder/23518), 301 (12665), 484 (14567), 519 nm (shoulder/10685 mol^–1^ dm^3^ cm^–1^); ^1^H NMR (600 MHz, (CD_3_)_2_CO, 25 °C) δ = 13.88 (s, 1H, C_ar_-O*H*-5), 12.61 (d, *J* = 12.6 Hz, 1H, C_ar_-O*H*-8), 12.06 (d, *J* = 15.3 Hz, 1H, C_ar_-O*H*-1), 9.64 (s, 1H, C_ar_-O*H*-7), 7.67 (s, 1H, C_ar_*H*-4), 7.15 (s, 1H, C_ar_*H*-2), 4.05 (s, 3H, OC*H*_3_-6), 2.50 ppm (s, 3H, C*H*_3_-3); MS (ESI, negative mode 4.5 kV) *m*/*z* (%) 315.0 (100) [M – H]^−^; *R*_*f*_ = 0.60 (stationary phase: SiO_2_; mobile phase: toluene–ethyl acetate–formic acid–acetic acid (70:20:5:5)).

### Singlet Oxygen Detection via the DMA Assay

The previously described DMA assay^56^ was employed to analyze the ability of four fungal extracts to generate singlet oxygen after irradiation. In brief, four stock solutions were prepared: a DMA solution in ethanol (*c* = 0.35 mM), a DMA solution (*c* = 0.35 mM) containing l-ascorbic acid (*c* = 5 mM) in ethanol, l-ascorbic acid (*c* = 5 mM) in ethanol, and pure ethanol (96%). The fungal extracts were dissolved in DMSO (*c* = 1 mg/mL), mixed with the stock solutions, and transferred into a 96-well plate, yielding a fungal extract concentration of *c* = 0.05 mg/mL per well. DMSO (*V* = 10 μL, 5%) was used as negative control; berberine (*c* = 0.15 mM) and Rose Bengal (RB) (*c* = 5 μM) were used as positive controls. Thereafter, the multiwell plates were irradiated by four cycles of blue light (λ = 468 nm, 1.24 J/cm^2^ min^1^, berberine = positive control) or green light irradiation (λ = 519 nm, 0.92 J/cm^2^ min^1^, RB = positive control). All measurements were done as technical duplicates. The results of the DMA assay were presented as the mean ± standard error. For the green light experiment, a slightly modified protocol was used, omitting the absorbance scan (λ = 200–600 nm) of each extract stock solution mixture at the five time points.

### Determination of the Singlet Oxygen Photoyield via NIR Measurements

The steady-state singlet oxygen emission measurements were performed on a previously reported custom-built setup utilizing a slightly modified experimental procedure.^59−61^ Perinaphthenone was used as a reference with reported φ_Δ_(^1^O_2_) = 0.98 ± 0.07 in air-saturated ACN and φ_Δ_(^1^O_2_) = 0.97 ± 0.04 in air-saturated CD_3_OD.^48,62^ Ru(bpy)_3_Cl_2_ was used as a validation compound with reported φ_Δ_(^1^O_2_) = 0.57 ± 0.06 in air-saturated ACN and φ_Δ_(^1^O_2_) = 0.73 ± 0.12 in air-saturated CD_3_OD.^49,63^ All the compounds were dissolved in ACN or CD_3_OD (*V* = 3 mL) and transferred into a macro fluorescence cuvette from Firefly (lightpaths: 1 cm × 1 cm). The irradiation of samples was done at 298 K using a 450 nm LRD-0450 Laserglow fiber-coupled laser set to 80 mW at the cuvette with help of a PM100USB Thorlabs power meter. The UV–vis and NIR spectra were recorded at 298 K with Agilent Cary 60 UV–vis and Avantes NIR256–1.7TEC spectrometers, respectively. The NIR emission spectra were acquired within 20 s. All the spectral data were processed with OriginPro 9.1 and Microsoft Office Excel 2016.

### GC-MS Analyses of the Sugar Residue

Spectral data of **3** pointed toward β-glucose-substituted **5**. To determine the absolute configuration of the glucose residue, a GC-MS analysis was carried out. A crude methanolic extract of *C. sanguineus* (*m* = 15.9 mg), an *n*-butanol fraction (*m* = 10.0 mg), and an ethyl acetate fraction (fraction M2, *m* = 3.8 mg) were separately dissolved in aqueous trifluoroacetic acid (TFA, *c* = 3 mol/L, *V* = 1 mL) and heated at *T* = 90 °C for 60 min. After cooling and the addition of water (*V* = 2 mL), the reaction mixture was extracted three times with ethyl acetate (*V* = 2 mL). The aqueous phase was dried under an air stream at room temperature and kept in a desiccator. d-Glucose (*m* = 1.0 mg, *n* = 5.5 μmol), l-glucose (*m* = 1.0 mg, *n* = 5.5 μmol), and the dried hydrolysate were derivatized with l-cysteine methyl ester hydrochloride (1.5 mg (8.7 μmol) in 200 μL of pyridine, *T* = 60 °C, *t* = 60 min), subsequently silylated with *N,O*-bis(trimethylsilyl)trifluoroacetamide and chlorotrimethylsilane (BSTFA:TMCS = 99:1, 200 μL, *T* = 60 °C, *t* = 60 min), and analyzed using GC-MS. GC-MS analysis was carried out on an Agilent 5975C Series GC/MSD system equipped with an Agilent 7693 autosampler and a Triple Axis detector (MS). An Agilent 19091S-433:1813.75629 HP-5MS 5% Phenyl Methyl Silox (325 °C: 30 m × 250 μm × 0.25 μm) column was used as stationary phase, and helium served as carrier gas. The oven temperature was first set to *T* = 170 °C. After the temperature was kept constant at *T* = 170 °C for 5 min, the oven was brought up to 270 °C with a heating rate of 3 °C/min. The oven was then heated to 320 °C at 20 °C/min and kept at this temperature for an additional 5 min. The total run time, injection volume, split ratio, and flow rate were set to *t* = 45.8 min, *V* = 1 μL, 50:1, and *Q* = 0.75 mL/min, respectively. The results of the GC-MS analysis are depicted in Figure S21.

### Microbial Strains and Cultivation

If not stated differently, all experiments were done as described previously.^50^ In brief, the strains used in this study were *C. albicans* (501670), *E. coli* (DSM1103), and *S. aureus* (DSM1104). The bacterial cultures were stored in darkness at *T* = 4 °C on LB agar. *C. albicans* was cultivated on PDA under the same conditions. For the PACT experiments, the stored cultures were reactivated, and an overnight culture was incubated (*T* = 37 °C, *t* = 15–18 h, dark conditions). Turbidity was adjusted to a McFarland standard of 0.5 to prepare the standard suspensions. For yeast suspensions, the turbidity was measured at λ = 530 nm. For bacteria suspensions, turbidity was measured at λ = 600 nm. Liquid media used for PACT experiments were Müller-Hilton broth for bacteria and RPMI-1640 (double strength) for yeast.

### PhotoMIC Assay

The photoantimicrobial experiments were performed as previously published.^50^ In short, two identical 96-well plates were prepared for dark and light treatment. On each plate, the extracts were tested (i.e., *c* = 5, 25, and 50 μg/mL), fraction blanks and medium blanks were measured, and the untreated population was recorded as growth control. If needed, the concentrations were adjusted to identify the break point. Curcumin, as an established photosensitizer, was utilized as a positive control. Bacterial cultures were adjusted via photometry to a 0.5 McFarland standard in water and diluted in a Müller-Hilton broth (1:50). Yeast suspensions adjusted to a 0.5 McFarland standard were diluted with RPMI-1640 (double strength, 1:10). Within 30 min after diluting the suspensions, the plates were inoculated (*V* = 50 μL) to reach (2–8) × 10^5^ CFU/mL for bacteria^64^ and (0.5–2.5) × 10^5^ CFU/mL for yeast.^65^ After 60 min of preincubation time in the dark, one plate was irradiated with a light dose of *H* = 30 J/cm^2^. The other plate was kept in the darkness at room temperature. Viability controls were drawn and plated on LB agar/PDA. The 96-well plates and LB agar/PDA plates were incubated at *T* = 37 °C in the dark. After 20–24 h, turbidity measurements were done after shaking for 15 seconds, followed by taking samples of wells that showed inhibition (>20%) of population growth control.

Assessment of the PACT experiment was done by correlating the treated wells to the uninhibited growth control. Turbidity of fraction-blank and medium-blank samples was subtracted from corresponding wells to eliminate deviation caused by darkening or bleaching of media and extracts. Each concentration of fungal extracts, the positive control, and the growth control was measured at least in triplicates.

### (Photo)cytotoxicity Assay

Cells of the adherent cancer cell lines A549 (non-small-cell lung cancer, ATCC, Sigma-Aldrich), AGS (stomach cancer, CLS, Eppelheim), and T24 (urinary bladder carcinoma, CLS, Eppelheim) were cultivated in 75 cm^2^ Nunc EasY flasks (product number: 51985042, 75 cm^2^) with Gibco MEMTM medium (product number: 42360081) supplemented with fetal calf serum (FCS, Biowest, S181BH-500, 10% v/v) and penicillin/streptomycin (P/S, 1% v/v, Gibco, R10378-016, final concentrations: 0.16 μg/mL streptomycin, 0.16 unit/mL penicillin). Cells were trypsinized every other day (confluency ∼80%) and used for 8–12 weeks. Freezing and thawing of cell cultures was done according to standard procedures. The (photo)cytotoxicity assay was performed as published elsewhere.^55,56^

Briefly, cells (AGS: 2500 cells/well, T24: 1500 cells/well, A549: 2000 cells/well) were seeded in 96-well plates in Gibco Opti-MEMTM (OMEM, product number: 11058021) containing FCS (2.4% v/v) and P/S (1% v/v) at 37 °C in a 5% CO_2_ atmosphere. The extracts/fractions were dissolved in DMSO (*c*_stock solution_ = 10 mg/mL) and further diluted with OMEM. The cells were treated 24 h after seeding with six different working solutions per extract/fraction. The final concentrations tested were 55.0, 27.5, 11.0, 5.5, 2.8, and 0.6 μg/mL for the extracts and 50.0, 25.0, 10.0, 5.0, 2.5, and 0.5 μg/mL for the fractions. Pure compounds were dissolved in DMSO (*c* = 5.0 mM) and diluted with OMEM to finally obtain test solutions with the following concentrations: 25.0, 12.5, 5.0, 2.5, 1.3, and 0.3 μM. After incubating the cells for an additional 24 h, the medium was aspirated and replaced by fresh OMEM (+ 2.4% v/v FCS, + 1% P/S). Of two identically treated plates one was irradiated for 7 min 30 s with blue light (λ = 468 ± 27 nm, *H* = 9.3 J/cm^2^)/22 min 23 s with green light (λ = 519 ± 33 nm, *H* = 30.0 J/cm^2^) and the other one was kept in the dark (light-independent/dark cytotoxicity). After the irradiation step, the plates were kept at 37 °C in a 5% CO_2_ atmosphere for another 48 h (total experiment time = 96 h). Then, the cells were fixed by careful addition of cold trichloroacetic acid (10% w/v in water, *V* = 100 μL/well) and stored in a refrigerator at 8 °C for at least 24 h. The fixed cell monolayers were washed with slow running deionized tap water and stained with sulforhodamin B (SRB) 0.4% w/v SRB in 1% v/v acetic acid, *V* = 100 μL/well) for 30 min. Thereafter, the plates were washed again (*n* = 5, 1% v/v acetic acid) and dried at room temperature. The dried dye was then redissolved in tris(hydroxymethyl)aminomethane solution (TRIS, 10 mM in water, *V* = 100 μL/well) and incubated for at least 20 min. Absorbance was measured at λ = 540 nm with a plate reader. EC_50_ values including their confidence intervals (95%) were calculated with GraphPad Prism 5 employing the relative Hill-slope equation [log(inhibitor) vs normalized response, variable slope]. The negative control was the illuminated, nontreated cells as well as the nonilluminated, nontreated cells. The photoindex (P.I.), which expresses the ratio of cells killed in the absence of light to cells killed after irradiation, was calculated as EC_50|dark_ divided by EC_50|irradiated_. Treated cells were microscopically investigated (Figures S22–S24).

## Supporting Information Available

Details of the fungal species collected, annotated chromatograms of the prepared extracts and dose-response curves of the (photo)antimicrobial experiments; several spectra (NMR, UV–Vis, IR) of the isolated compounds **3** and **5**; and micrographs of the treated cells (PDF)
