# Supplementary material for: Fungal Anthraquinone Photoantimicrobials Challenge the Dogma of Cationic Photosensitizers
Source: J Nat Prod. 2023 Sep 14;86(10):2247–57. doi: 10.1021/acs.jnatprod.2c01157 (PMC10616806; doi:10.1021/acs.jnatprod.2c01157)
Supplement: Supplementary file 1 — np2c01157_si_001.pdf [file np2c01157_si_001.pdf]

Supplementary Part

Fungal Anthraquinone Photoantimicrobials Challenge the Dogma of Cationic Photosensitizers

Fabian Hammerle[#,†], Johannes Fiala[#,†], Anja Höck[†], Lesley Huymann[†,‡], Pamela Vrabl[‡], Yurii Husiev[§], Sylvestre Bonnet[§], Ursula Peintner[‡], and Bianka Siewert[†,*]

[†] Department of Pharmacognosy, Institute of Pharmacy, University of Innsbruck, Innsbruck, Austria

[‡] Institute of Microbiology, University of Innsbruck, Innsbruck, Austria

[§]Leiden Institute of Chemistry, Leiden University, Leiden, The Netherlands

#These authors contributed equally

Table S 1 Collection details, Voucher number, and GenBank details of the investigated fungi

Full name	GenBank / Voucher	Collection date	Origin	Leg. et det.
Cortinarius sanguineus var. *aurantiovaginatus* Fillion & Moenne-Locc.	OL712403 / IBF20200072	02.10.2020	Mutters, Tirol, Austria	L. Huymann
Cortinarius holoxanthus (M.M. Moser & I. Gruber) Nezdojm.	OL712405 / IBF20200070	28.09.2020	Natters, Tirol, Austria	L. Huymann
Cortinarius cinnabarinus Fr.	OM638753 /IBF20190153	28.09.2019	Campo Felice, L'Aquila, Italy	M. Leonardi
Cortinarius malicorius Fr.	OL712404 / IBF20200071	09.10.2020	Natters, Tirol, Austria	L. Huymann

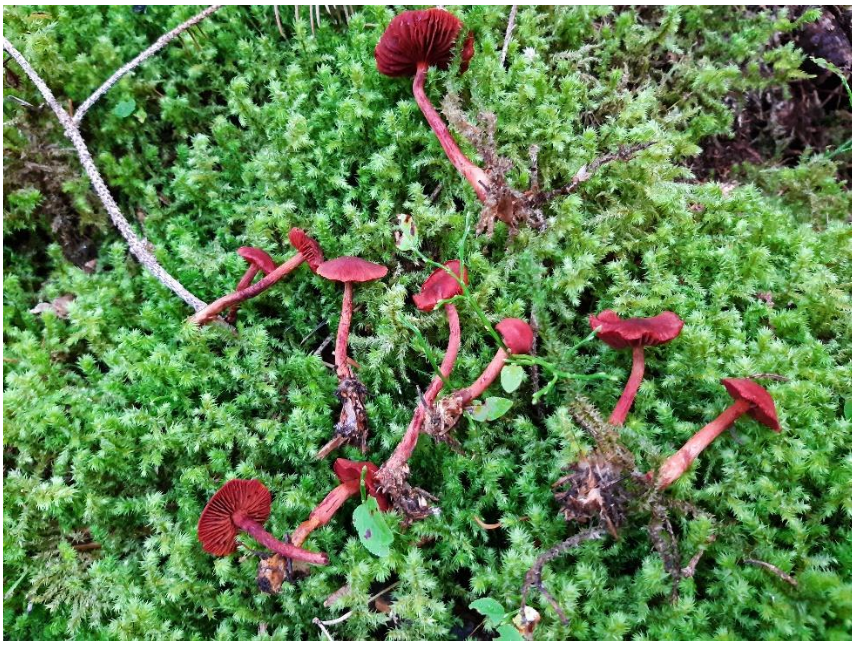

Figure S 1 in situ photograph of the *C. sanguineus* collection (IBF20200072)

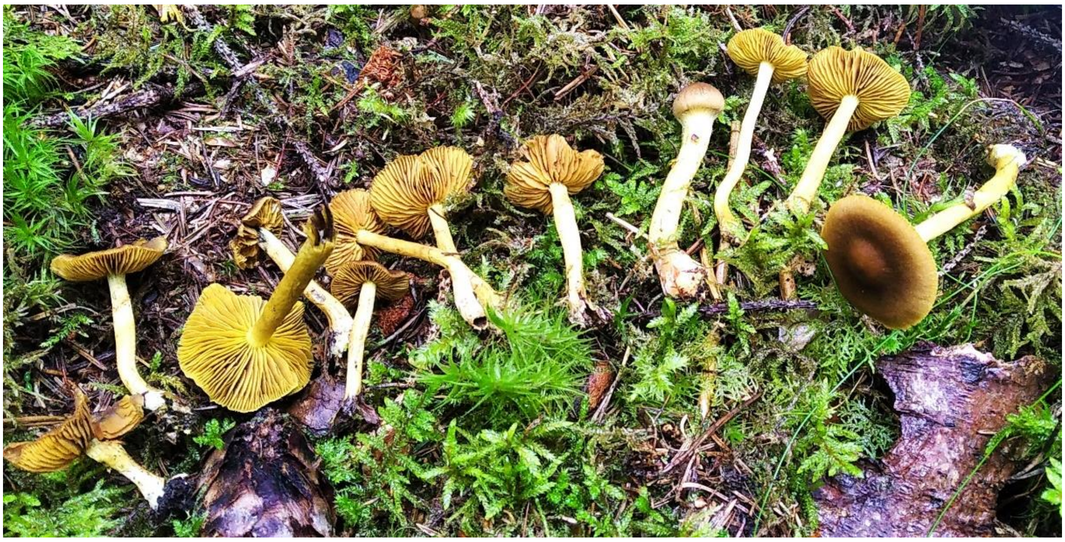

Figure S 2. In situ picture of the *C. holoxanthus* collection (IBF20200070)

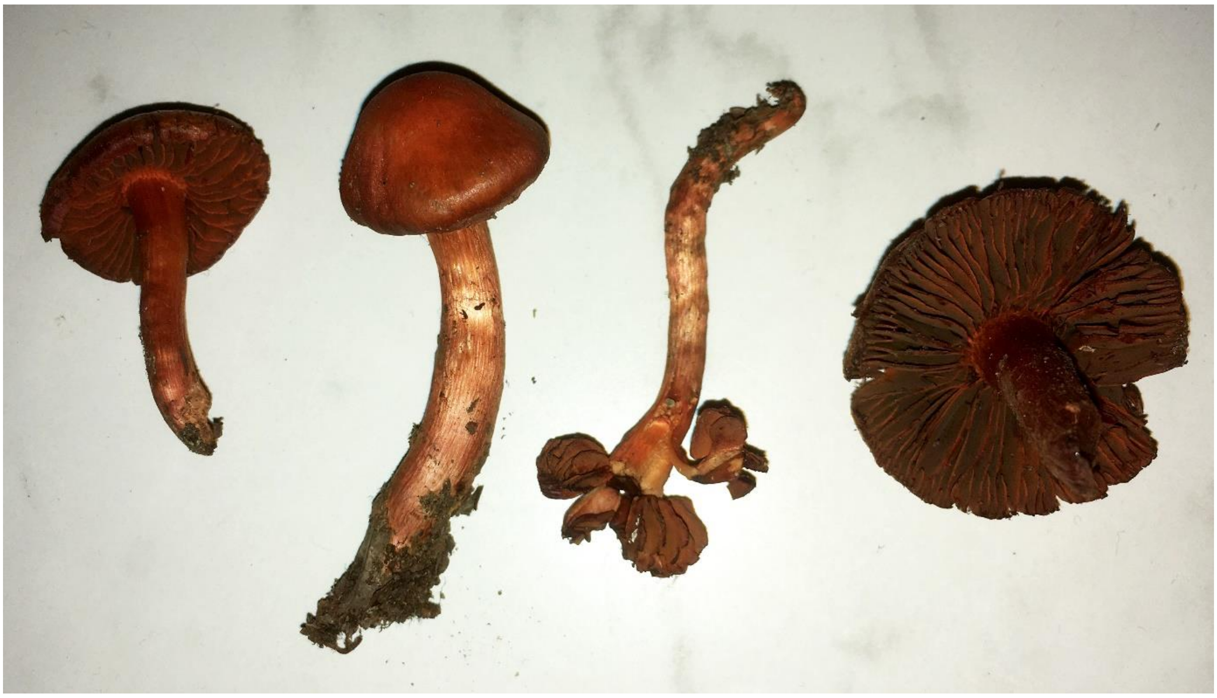

Figure S 3. Photograph of *C. cinnabarinus* (IBF20190153)

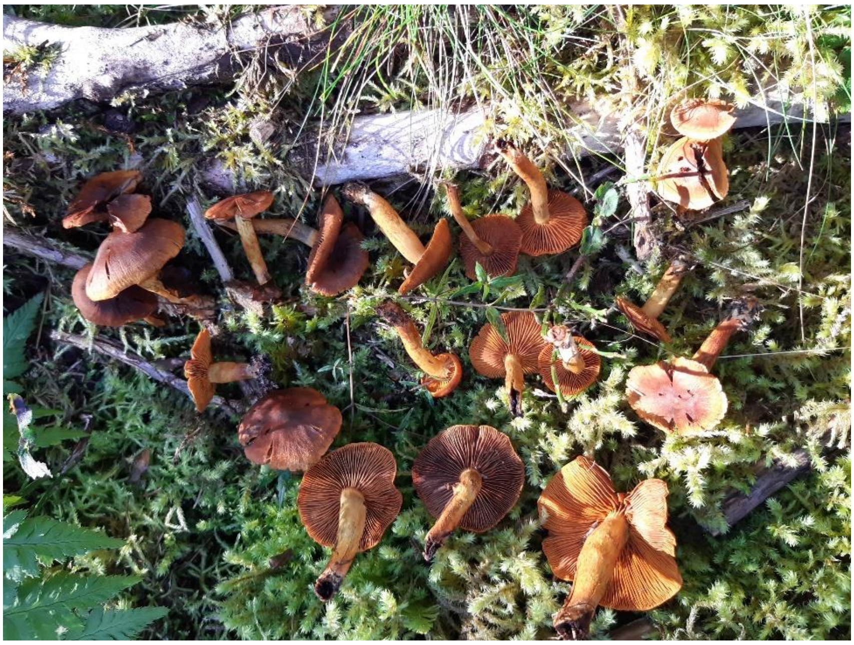

Figure S 4. In situ picture of the *C. malicorius* collection (IBF20200071)

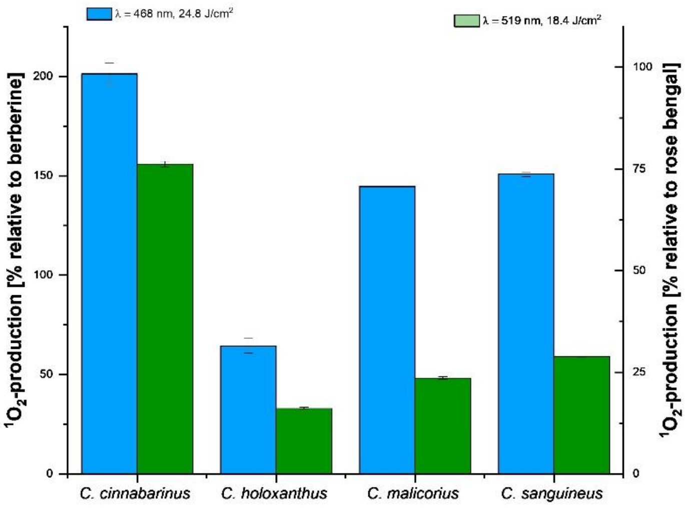

Figure S 5. Results of the DMA-assay probing for the in-situ production of singlet oxygen. The singlet oxygen production is given relative to the utilized positive control (i.e., berberine for blue light (λ = 468 nm, H = 24.8 J/cm²) and rose bengal for green light (λ = 519 nm, H = 18.4 J/cm²) irradiation)

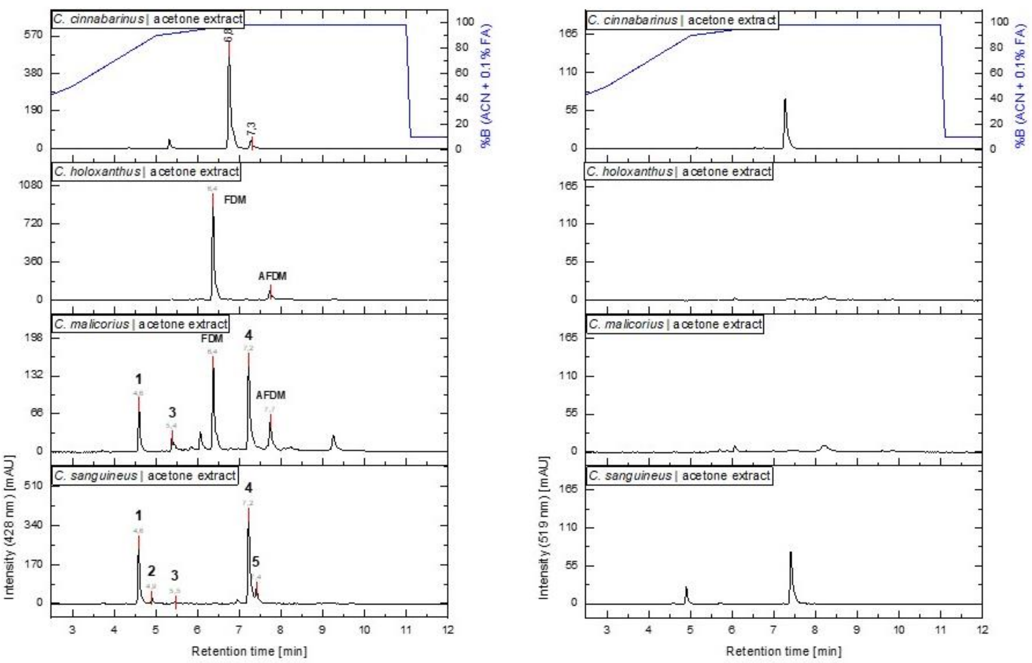

Figure S 6. HPLC-DAD Chromatograms (left: $\lambda_{detection}$ = 428 nm, right: $\lambda_{detection}$ = 519 nm) of the four different fungal extracts. The HPLC analysis was performed using a Phenomenex Synergi MAX-RP 80 Å (150 x 4.60 mm, 4 micron) column as stationary phase at T = 40 °C. The mobile phase consisted of water (A) and acetonitrile with 0.1% formic acid (B). The flow rate was adjusted to 1.0 mL/min. The applied gradient is indicated by the blue line (top). The isolated pigments were annotated with numbers. Wheree meaningful, the obtained peaks were annotated based on their UV-Vis properties, MS-Peak, and in comparison to authentic reference compounds.

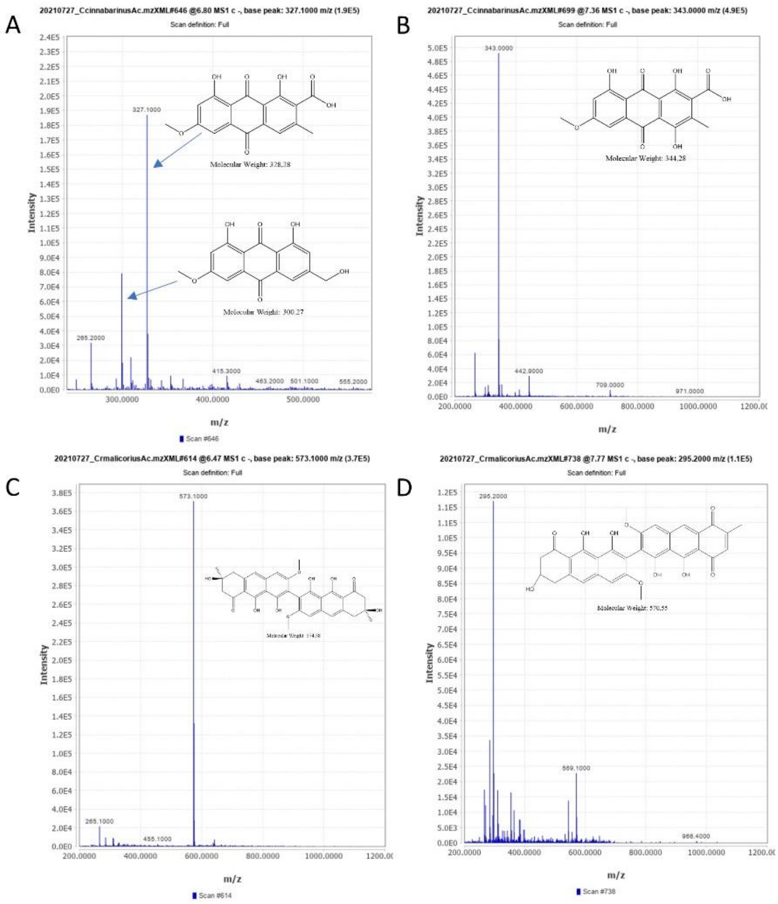

Figure S 7. MS-Traces of the annotated pigments from C. cinnabarinus [**A**) retention time (rt) = 6.8 min, cinnalutein and fallacinol, **B**) rt = 7.3 min, cinnarubin] and C. rubrophyllus or C. holoxanthus [**C**) rt = 6.4, flavomannin-6,6’-dimethylether (FDM), **D**) rt = 7.7 min, anhydroflavomannin-9,10-chinon-6,6´-di-O-methylether (AFDM)].

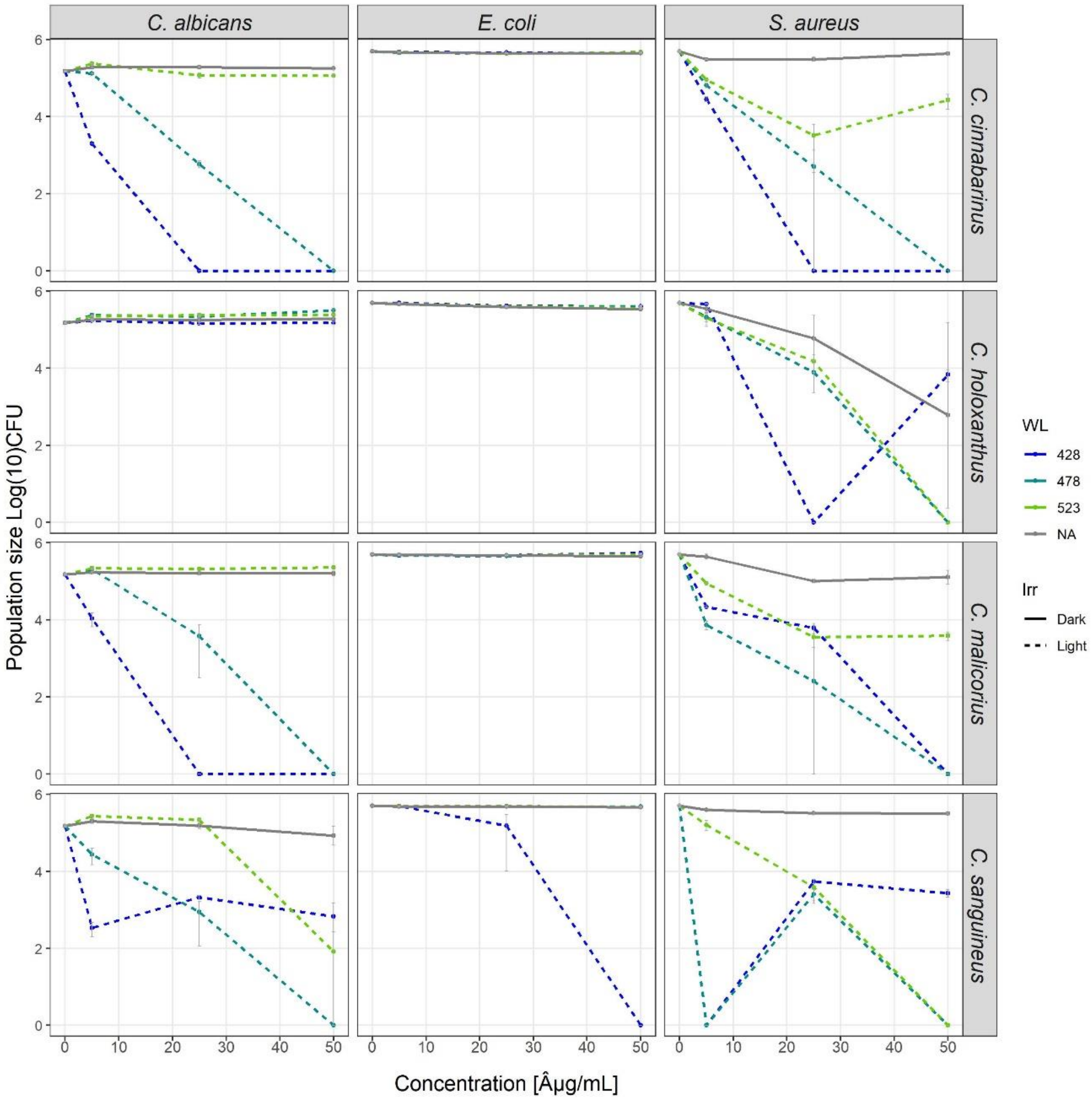

Figure S8. Dose-response curves, plotting the logarithm of the CFUs against the tested concentrations, of the extracts against the three tested microorganisms under irradiation (dotted lines, violet, blue, and green, all 30 J/cm²) as well as in the dark. WL = Wavelength, Irr = Irradiation condition.

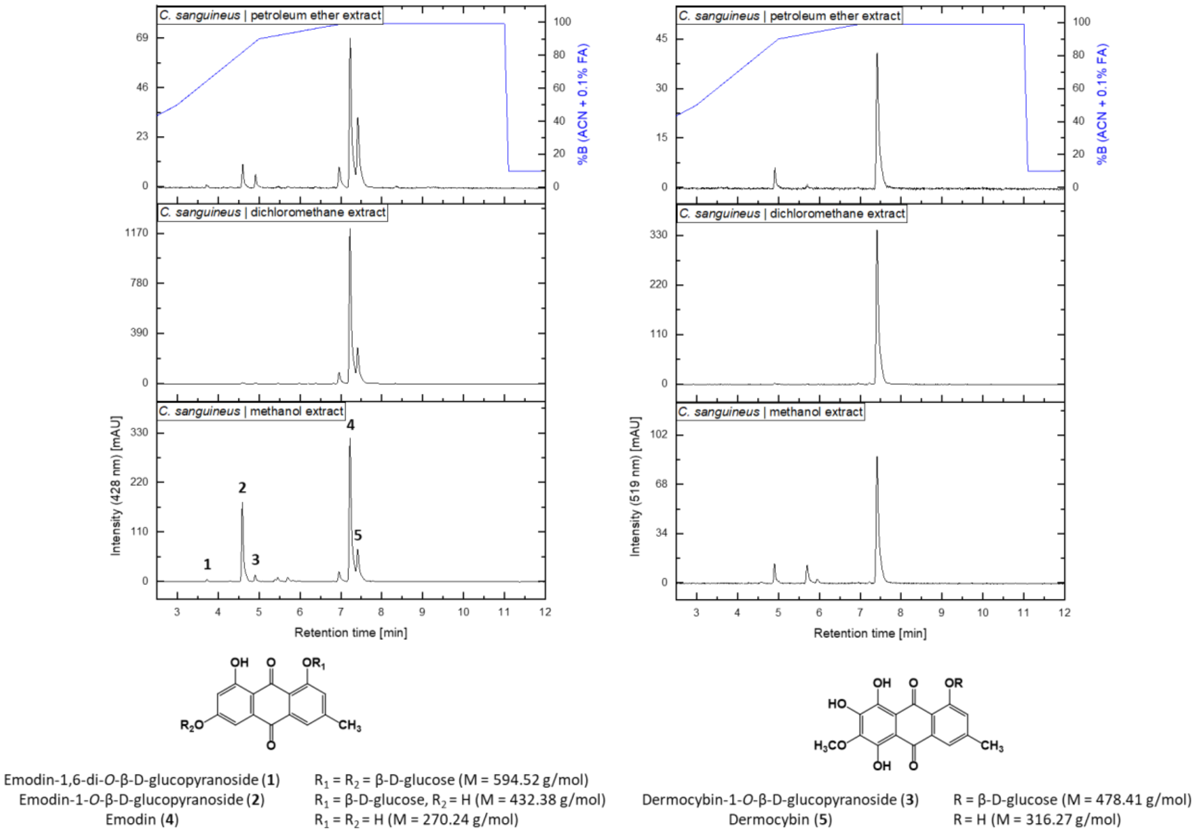

Figure S9. Chromatograms (left: $\lambda_{detection}$ = 428 nm, right: $\lambda_{detection}$ = 519 nm) of the petroleum ether, dichloromethane, and methanol extract of *C. sanguineus* fruiting bodies (c = 1.0 mg/mL, DMSO). The pigments investigated in this study were highlighted with numbers above their respective peaks (chromatogram bottom left). The numbers correspond to the chemical structures on the bottom. The HPLC analysis was performed using a Phenomenex Synergi MAX-RP 80 Å (150 x 4.60 mm, 4 micron) column as stationary phase at T = 40 °C. The mobile phase consisted of water (A) and acetonitrile with 0.1% formic acid (B). The flow rate was adjusted to 1.0 mL/min. The applied gradient is indicated by the blue line (top).

Table S2. List of detected secondary metabolites with their respective retention times and molecular masses (HPLC-DAD-MS experiment: negative ionisation mode).

**Name**	**Compound #**	**Retention time [min]**	**m/z**
Emodin-1,6-di-*O*-β-D-glucopyranoside	**1**	3.83	593.2 $[M-H]^-$, 431.0 $[M-C_6H_{10}O_5$ (1 glucopyranoside residue)$]^-$, 269.1 $[M- C_{12}H_{20}O_{10}$ (2 glucopyranoside residues)$]^-$
Emodin-1-*O*-β-D-glucopyranoside	**2**	4.69	431.0 $[M-H]^-$
Dermocybin-1-*O*-β-D-glucopyranoside	**3**	5.00	477.1 $[M-H]^-$
Emodin	**4**	7.32	269.0 $[M-H]^-$
Dermocybin	**5**	7.52	315.0 $[M-H]^-$

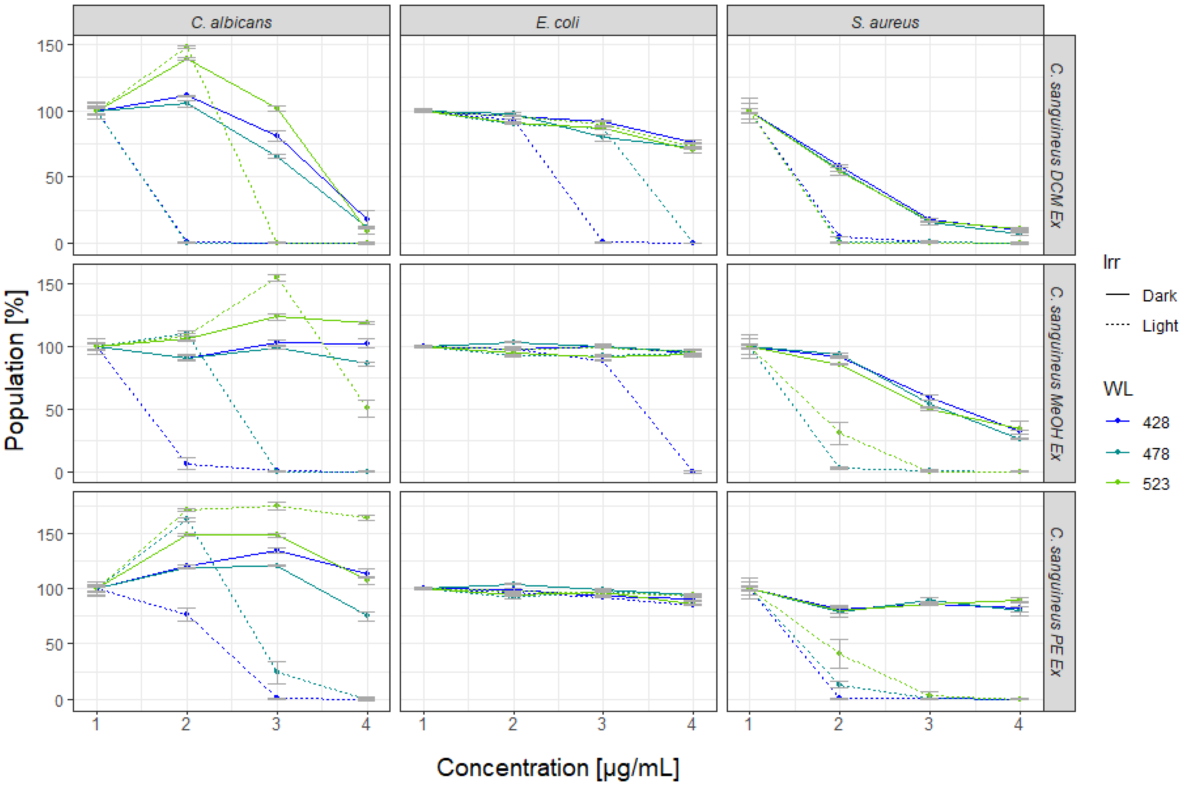

Figure S 10. Dose-response curves obtained from the photoantimicrobial assay. The suspensions of the microorganisms (Candida albicans, Escherichia coli, and Staphylococcus aureus) were preincubated for t = 10 min and irradiated with the indicated wavelengths (H = 30 J cm^{-2}).

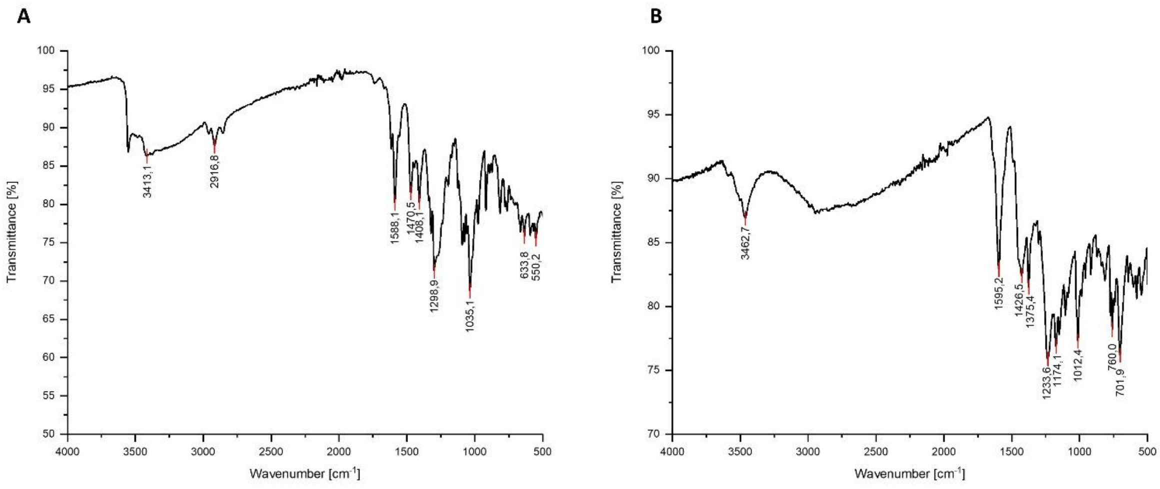

Figure S11. A) IR-spectrum of dermocybin-1-*O*-β-D-glucopyranoside (**3**) and **B**) dermocybin (**5**).

1.1.1 UV/Vis-spectra

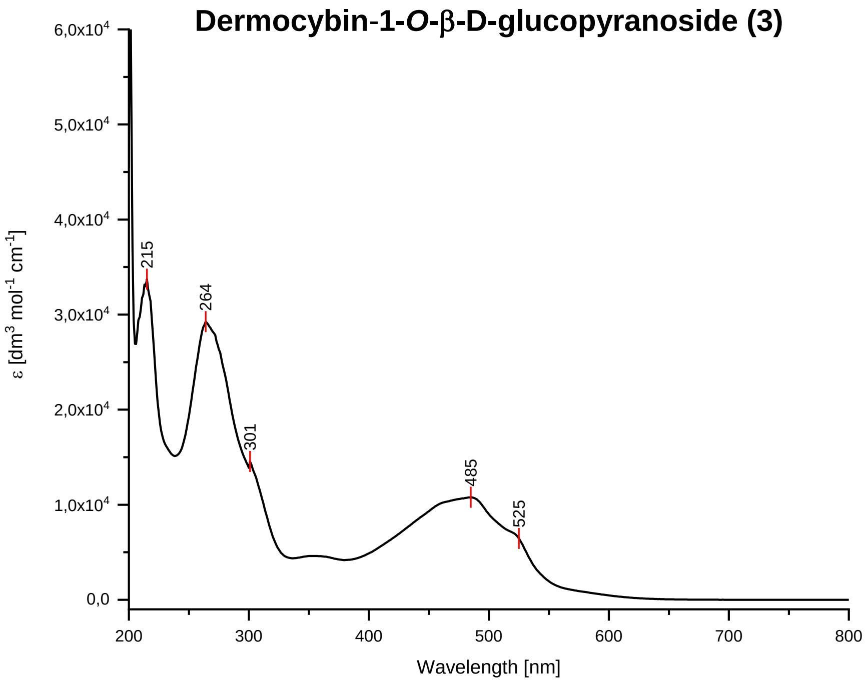

Figure S12. UV/Vis-spectrum of dermocybin-1-*O*-β-D-glucopyranoside (**3**) in MeOH.

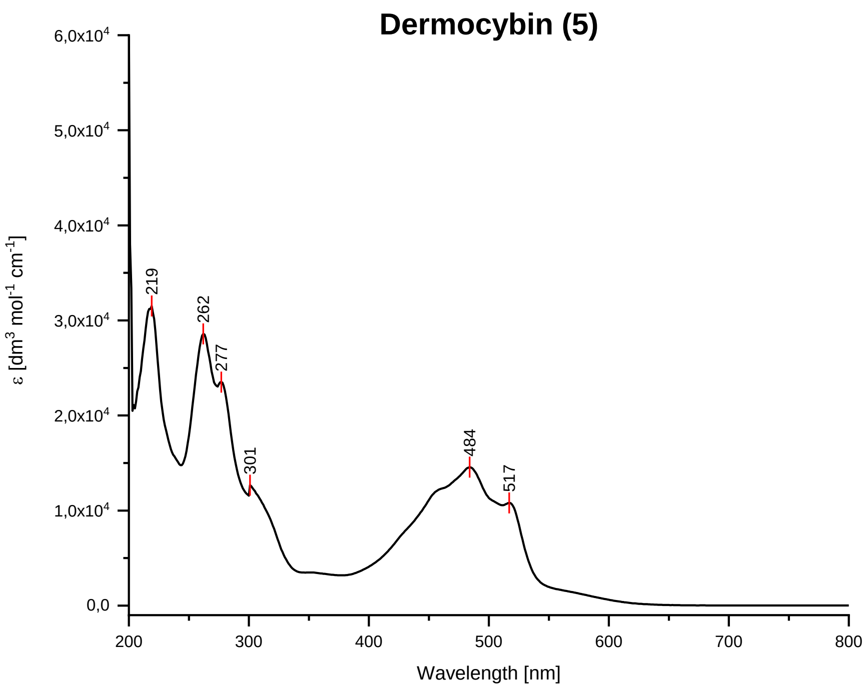

Figure S13. UV/Vis-spectrum of dermocybin (**5**) in MeOH.

1.1.2 NMR-spectra

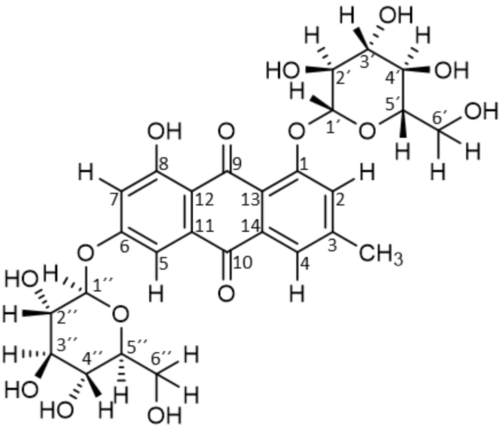

Emodin-1,6-di-*O*-β-D-glucopyranoside (1)

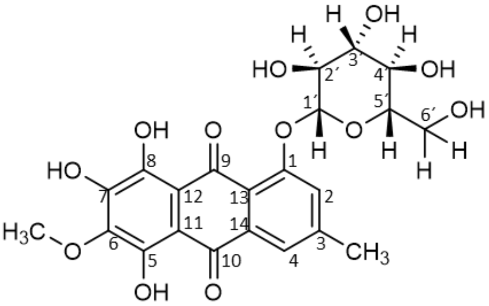

Dermocybin-1-*O*-β-D-glucopyranoside (3)

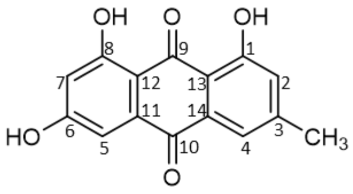

Emodin (4)

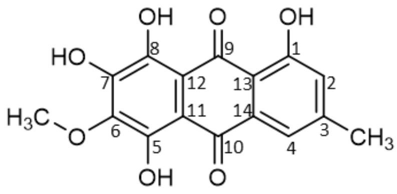

Dermocybin (5)

Figure S14. Numbering used for the isolated anthraquinones (adopted from [*Danielsen, K., D.W. Aksnes, and G.W. Francis,* NMR study of some anthraquinones from rhubarb. *Magnetic Resonance in Chemistry, 1992.* ***30****(4): p. 359-360*]).

1.1.2.1 NMR spectral data (tabular form)

Table S3. ^{13}C NMR (150 MHz) and ^{1}H NMR (600 MHz) chemical shifts for **3** (Methanol-d4) and **5** (Acetone-d6). ^{13}C values for **3** and **5** were mainly based on HSQC and HMBC experiments. Abbreviations: n.o. … not observed, - … not present.

	Dermocybin-1-*O*-β-D-glucopyranoside (**3**) (Methanol-d4)		Dermocybin (**5**) (Acetone-d6)	
C	δ C (ppm)	δ H (ppm)	δ C (ppm)	δ H (ppm)
1	160.2	-	163.8	12.06 OH, *s*
2	125.0	7.59 *s*	124.7	7.15 *s*
3	149.0 C_{ar} | 22.3 CH_3	2.53 *s*	150.5 C_{ar} | 22.5 CH_3	2.50 *s*
4	123.6	7.90 *s*	121.2	7.67 s
5	n.o.	n.o.	156.3	13.88 *s*
6	142.2 C_{ar} | 61.0 OCH_3	4.02 OCH_3, *s*	143.0 C_{ar} | 61.4 OCH_3	4.05 OCH_3, *s*
7	n.o.	n.o.	148.8	9.64 OH, *s*
8	n.o.	n.o.	150.7	12.61 OH, *s*
9	n.o.	-	n.o.	-
10	184.6	-	186.1	-
11	n.o.	-	107.1	-
12	n.o.	-	109.7	-
13	120.8	-	114.6	-
14	n.o.	-	n.o.	-
1´	104.3	5.06 *d*		
2´	75.3	3.72 – 3.65 *m*		
3´	77.5	3.61 – 3.54 *m*		
4´	71.6	3.49 – 3.44 *m*		
5´	78.9	3.61 – 3.54 *m*		
6´	63.1	a: 3.99 *dd*<br>b: 3.77 *dd*		

1.1.2.2 Dermocybin-1-*O*-β-D-glucopyranoside (3)

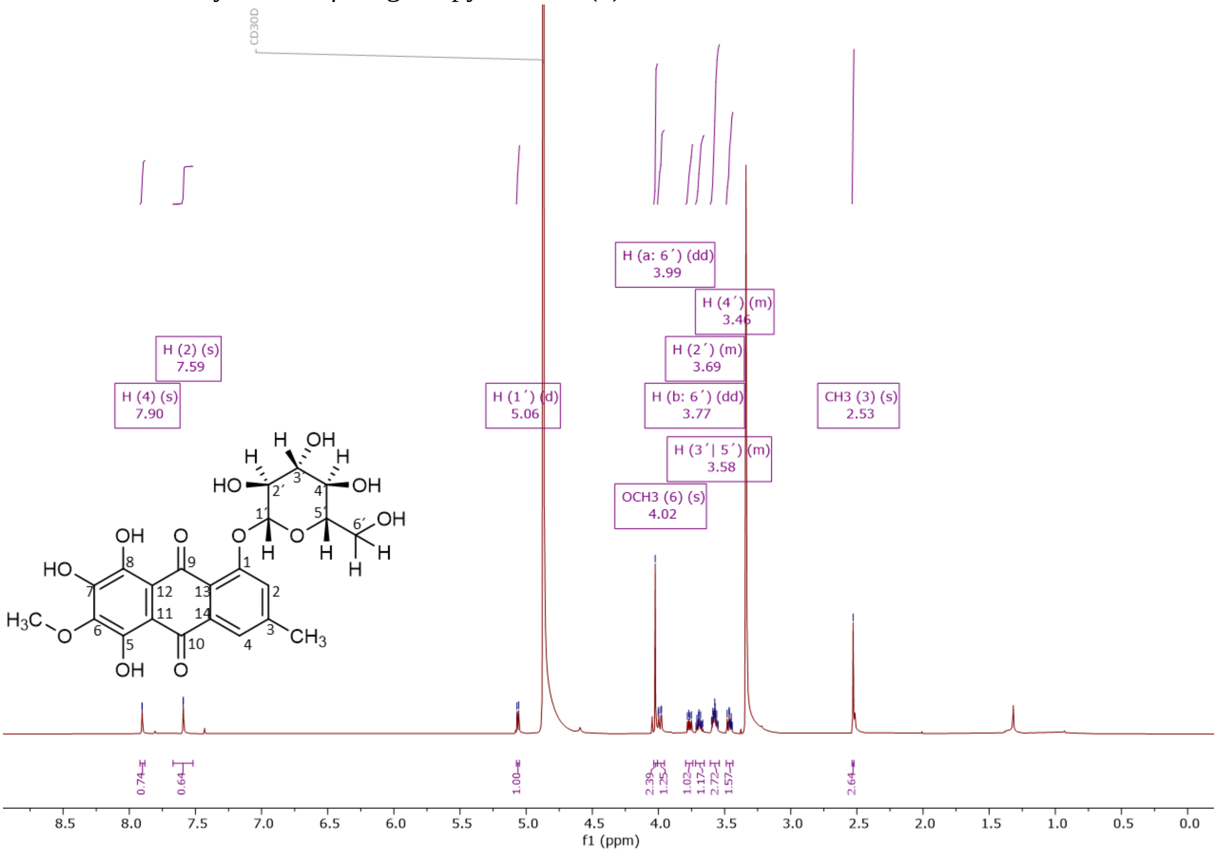

Figure S15. ^{1}H-NMR (600.19 MHz, Methanol-d4, 25 °C): Dermocybin-1-*O*-β-D-glucopyranoside (**3**).

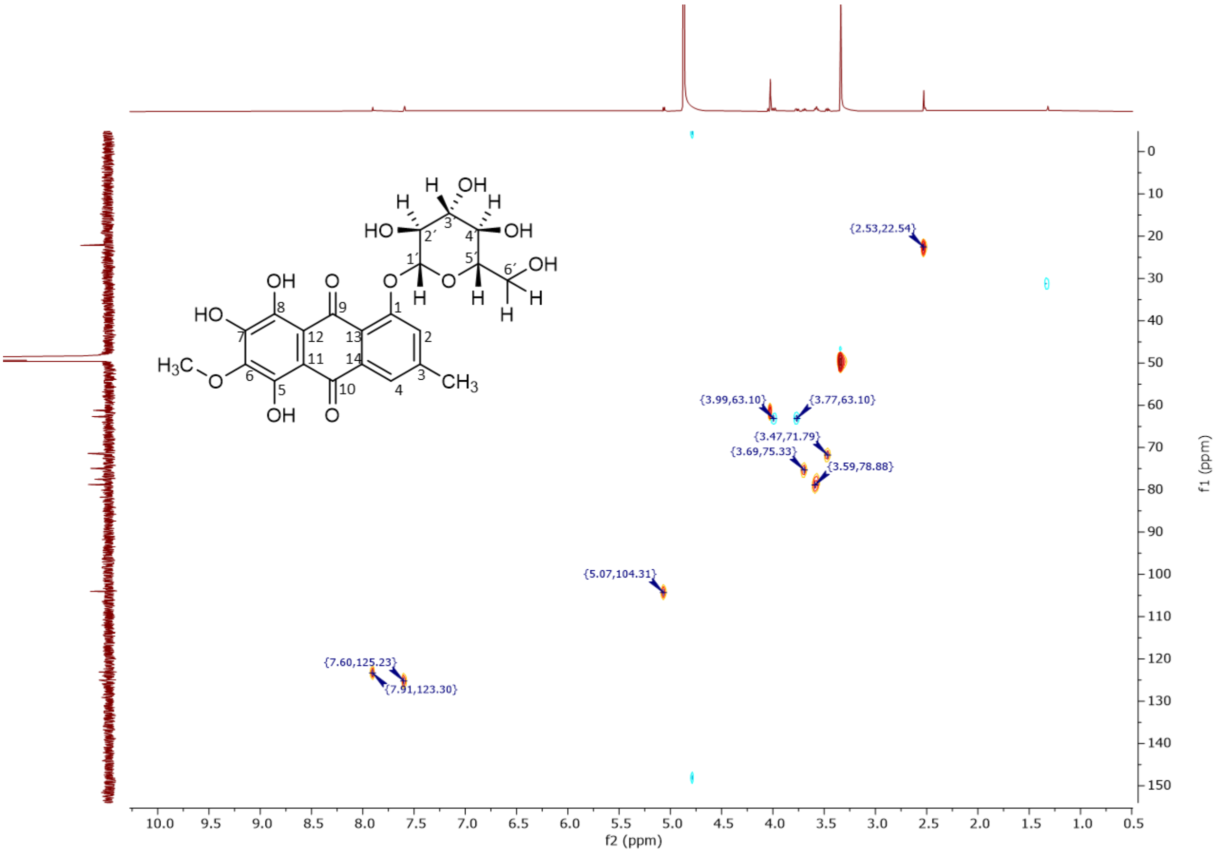

Figure S16. HSQC (600/150 MHz, Methanol-d4, 25 °C): Dermocybin-1-*O*-β-D-glucopyranoside (**3**).

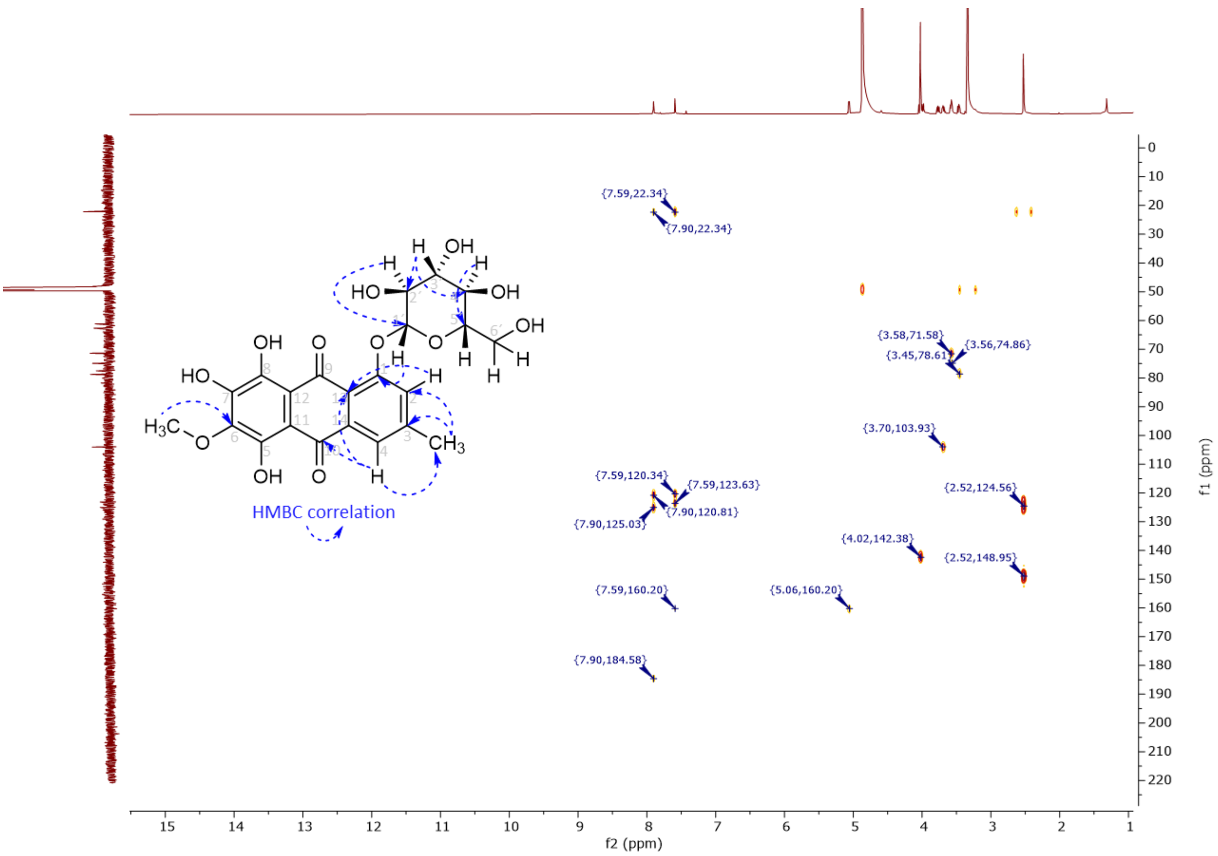

Figure S17. HMBC (600/150 MHz, Methanol-d4, 25 °C): Dermocybin-1-*O*-β-D-glucopyranoside (**3**).

1.1.2.3 Dermocybin (**5**)

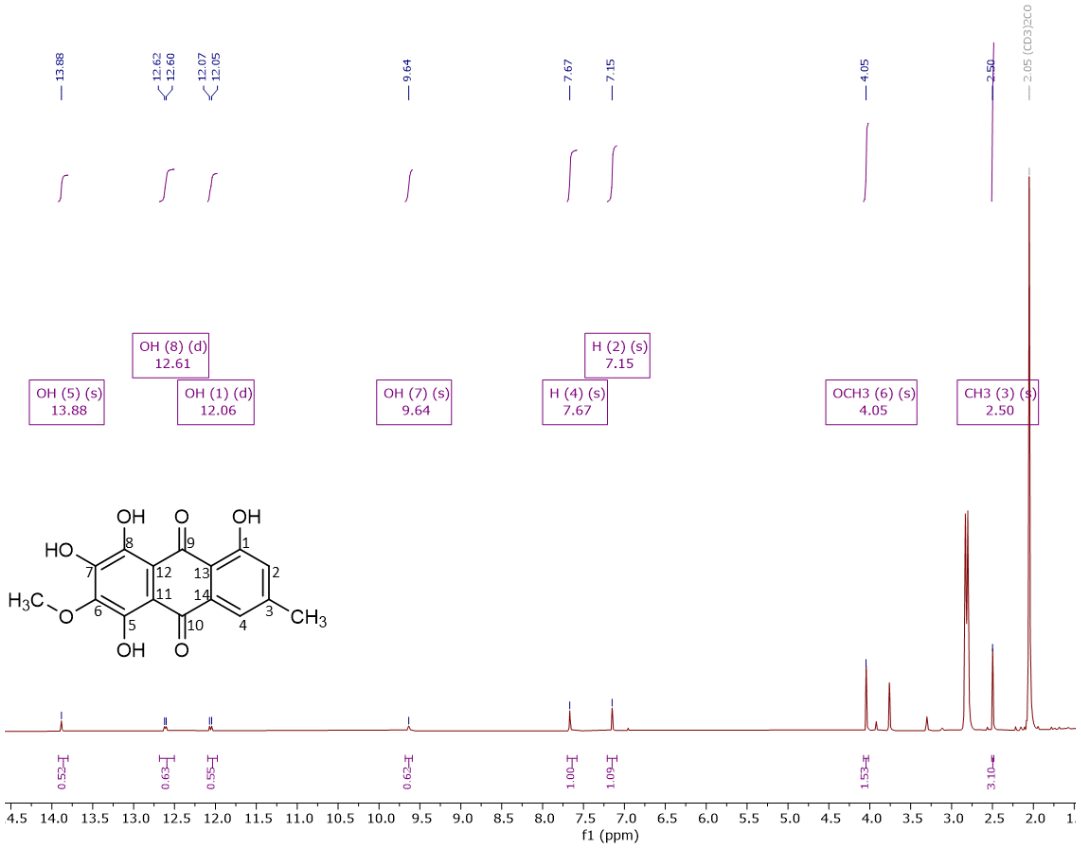

Figure S18. ^{1}H-NMR (600.19 MHz, Acetone-d6, 25 °C): Dermocybin (**5**).

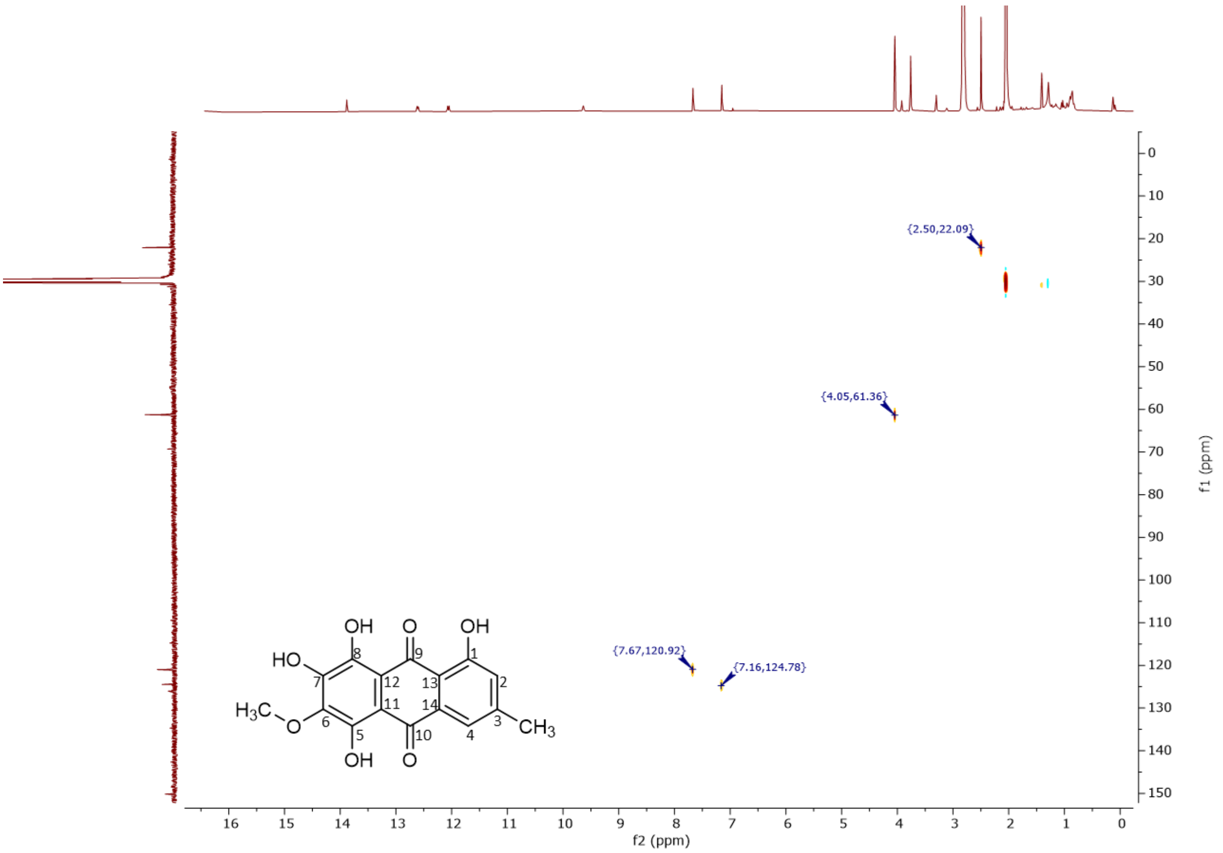

Figure S19. HSQC (600/150 MHz, Acetone-d6, 25 °C): Dermocybin (**5**).

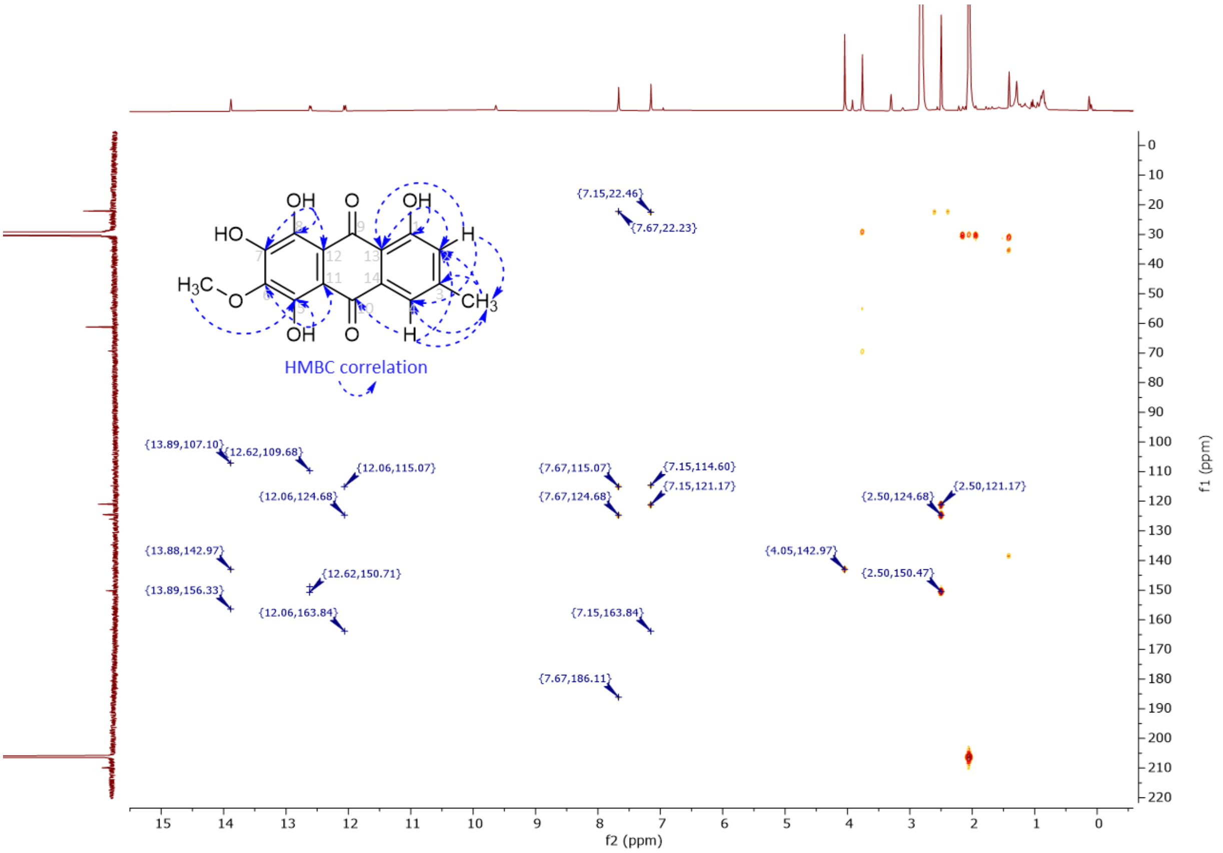

Figure S20. HMBC (600/150 MHz, Acetone-d6, 25 °C): Dermocybin (**5**).

1.2 GC-MS analysis

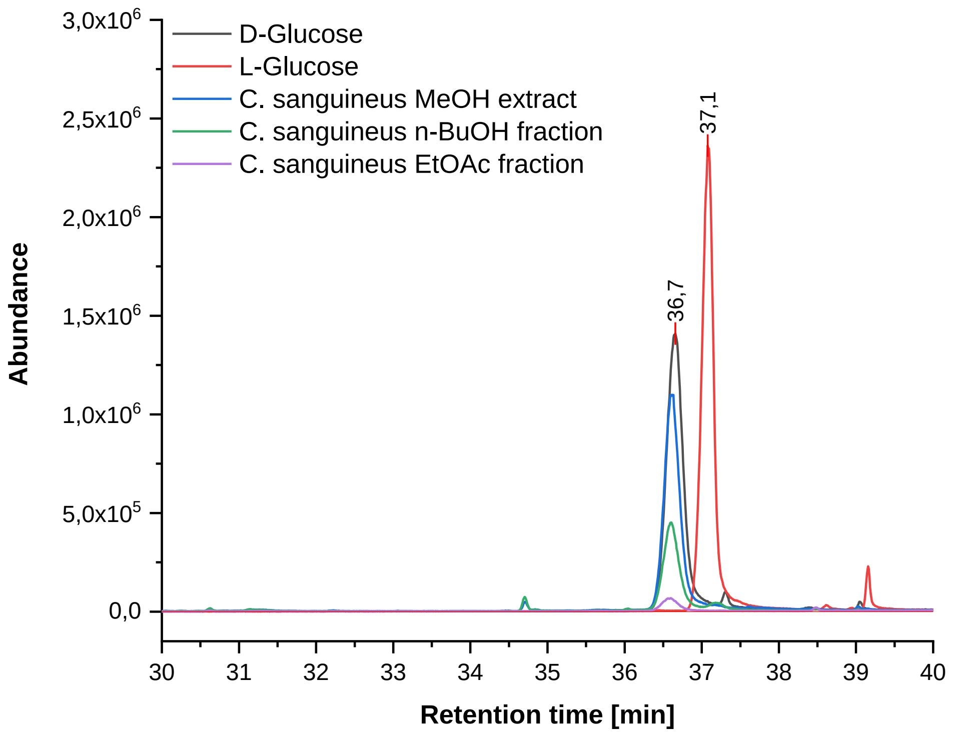

Figure S21. Results of the GC-MS analysis of the hydrolyzed *C. sanguineus* methanol extract as well as fractions for the determination of the sugar moiety present in **3**. The chromatogram displays the thiazolidine derivatives of the extract/fractions of *C. sanguineus* (MeOH extract: blue line, n-butanol/n-BuOH fraction: green line, ethyl acetate/EtOAc fraction: purple line), of L-Glucose (red line), and of D-glucose (black line) after derivatisation with BSTFA. The peaks representing the derivates of D-glucose and L-glucose showed retention times of 36.7 min and 37.1 min, respectively.

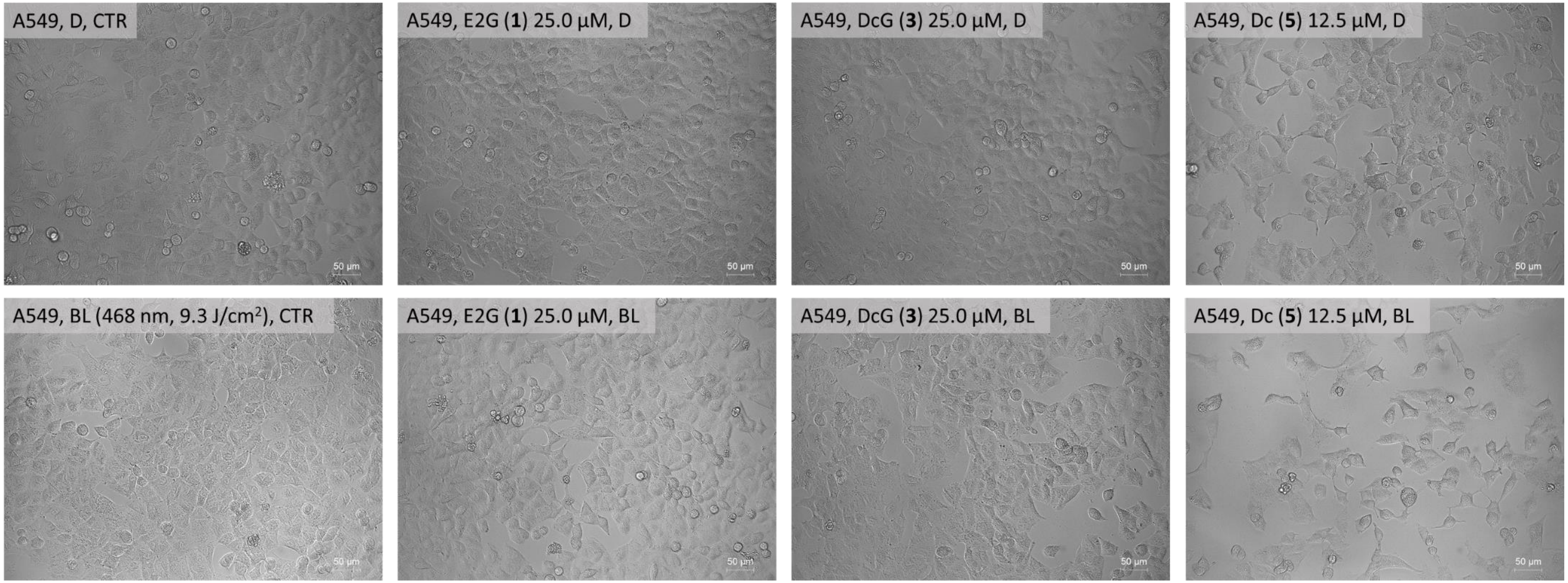

Figure S22. Micrographs of cells of the lung cancer cell line (A549 / human Caucasian lung carcinoma, 20x objective) treated (24 h) with emodin-1,6-di-*O*-β-D-glucopyranoside (E2G, **1**, c = 25.0 µM), dermocybin-1-*O*-β-D-glucopyranoside (DcG, **3**, c = 25.0 µM), and dermocybin (Dc, **5**, c = 12.5 µM). The upper line of pictures shows treated cells in the dark, the lower after irradiation with blue light (468 nm, 9.3 J/cm²).

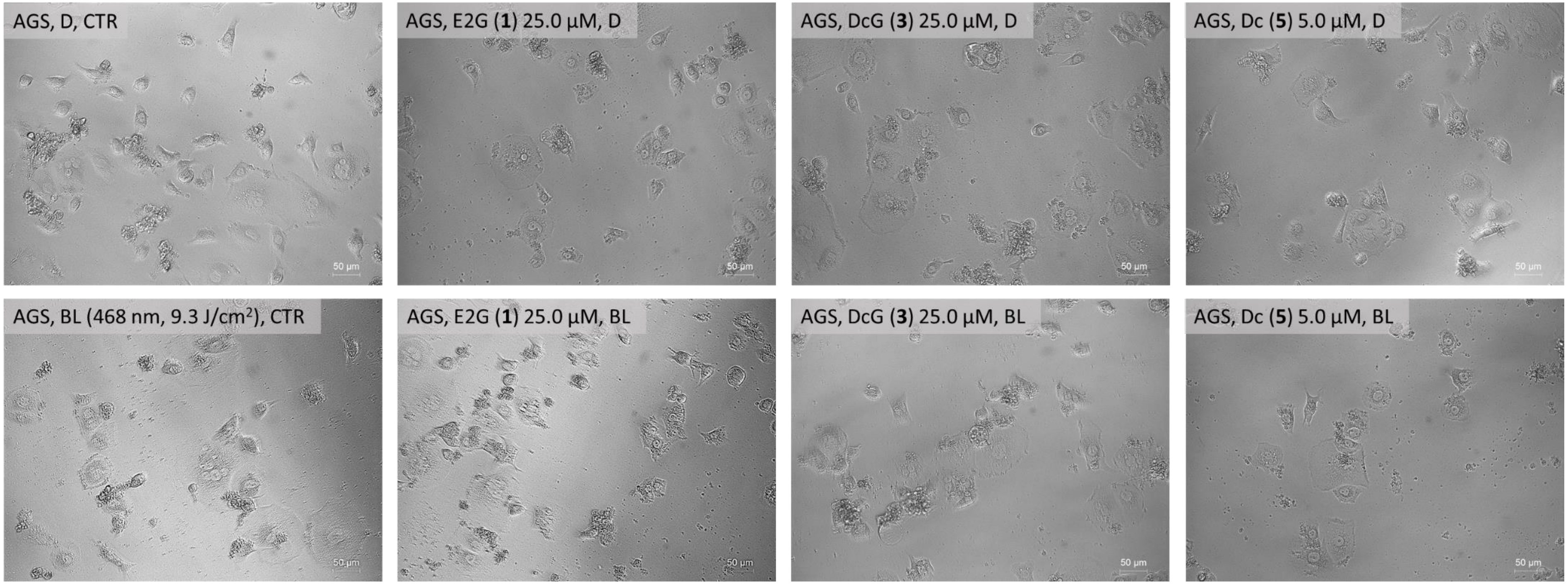

Figure S23. Micrographs of cells of the stomach cancer cell line (AGS / human Caucasian gastric adenocarcinoma, 20x objective) treated (24 h) with emodin-1,6-di-*O*-β-D-glucopyranoside (E2G, **1**, c = 25.0 µM), dermocybin-1-*O*-β-D-glucopyranoside (DcG, **3**, c = 25.0 µM), and dermocybin (Dc, **5**, c = 5.0 µM). The upper line of pictures shows treated cells in the dark, the lower after irradiation with blue light (468 nm, 9.3 J/cm²).

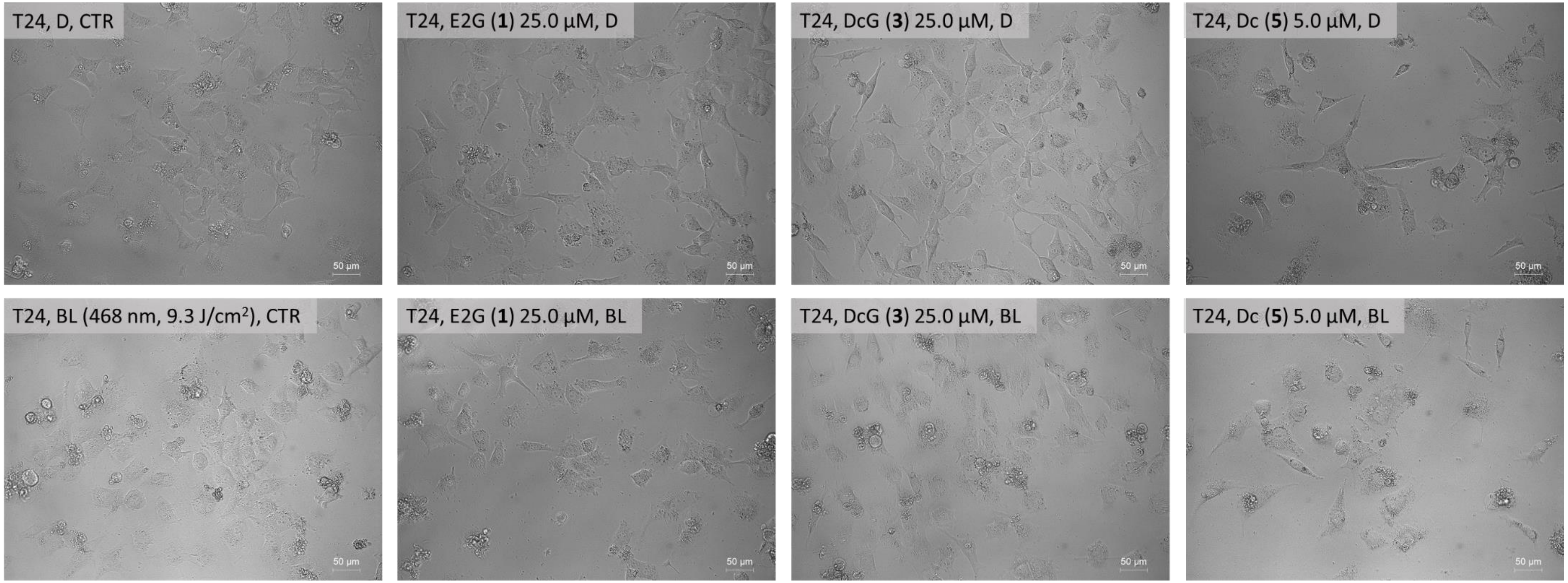

Figure S24. Micrographs of cells of the bladder cancer cell line (T24 / human bladder carcinoma, 20x objective) treated (24 h) with emodin-1,6-di-*O*-β-D-glucopyranoside (E2G, **1**, c = 25.0 µM), dermocybin-1-*O*-β-D-glucopyranoside (DcG, **3**, c = 25.0 µM), and dermocybin (Dc, **5**, c = 5.0 µM). The upper line of pictures shows treated cells in the dark, the lower after irradiation with blue light (468 nm, 9.3 J/cm²).
